# Rapid response systems, antibiotic stewardship and medication reconciliation: a scoping review on implementation factors, activities and outcomes

**DOI:** 10.1136/bmjqs-2024-017185

**Published:** 2024-06-06

**Authors:** Jonas Torp Ohlsen, Eirik Søfteland, Per Espen Akselsen, Jörg Assmus, Stig Harthug, Regina Küfner Lein, Nick Sevdalis, Hilde Valen Wæhle, John Øvretveit, Miriam Hartveit

**Affiliations:** 1 Department of Anaesthesia and Intensive Care, Haukeland University Hospital, Bergen, Norway; 2 Department of Clinical Medicine, University of Bergen, Bergen, Norway; 3 Norwegian Centre for Antibiotic use in Hospitals, Haukeland University Hospital, Bergen, Norway; 4 Centre for Clinical Research, Haukeland University Hospital, Bergen, Norway; 5 University of Bergen, Bergen, Norway; 6 Centre for Behavioural and Implementation Science Interventions, Yong Loo Lin School of Medicine, National University of Singapore, Singapore; 7 Department of Research and Development, Haukeland University Hospital, Bergen, Norway; 8 Department of Global Public Health and Primary Care, University of Bergen, Bergen, Norway; 9 Medical Management Center, Karolinska Institute, Stockholm, Sweden; 10 Fonna Hospital Trust, Haugesund, Norway

**Keywords:** Antibiotic management, Medical emergency team, Medication reconciliation, Implementation science, Patient safety

## Abstract

**ABSTRACT:**

**Introduction:**

Many patient safety practices are only partly established in routine clinical care, despite extensive quality improvement efforts. Implementation science can offer insights into how patient safety practices can be successfully adopted.

**Objective:**

The objective was to examine the literature on implementation of three internationally used safety practices: medication reconciliation, antibiotic stewardship programmes and rapid response systems. We sought to identify the implementation activities, factors and outcomes reported; the combinations of factors and activities supporting successful implementation; and the implications of the current evidence base for future implementation and research.

**Methods:**

We searched Medline, Embase, Web of Science, Cumulative Index to Nursing and Allied Health Literature, PsycINFO and Education Resources Information Center from January 2011 to March 2023. We included original peer-reviewed research studies or quality improvement reports. We used an iterative, inductive approach to thematically categorise data. Descriptive statistics and hierarchical cluster analyses were performed.

**Results:**

From the 159 included studies, eight categories of implementation activities were identified: *education; planning and preparation; method-based approach; audit and feedback; motivate and remind; resource allocation; simulation and training;* and *patient involvement*. Most studies reported activities from multiple categories. Implementation factors included: c*linical competence and collaboration; resources; readiness and engagement; external influence; organisational involvement; QI competence;* and *feasibility of innovation*. Factors were often suggested post hoc and seldom used to guide the selection of implementation strategies. Implementation outcomes were reported as: *fidelity or compliance; proxy indicator for fidelity; sustainability; acceptability;* and *spread*. Most studies reported implementation improvement, hindering discrimination between more or less important factors and activities.

**Conclusions:**

The multiple activities employed to implement patient safety practices reflect mainly method-based improvement science, and to a lesser degree determinant frameworks from implementation science. There seems to be an unexploited potential for continuous adaptation of implementation activities to address changing contexts. Research-informed guidance on how to make such adaptations could advance implementation in practice.

**WHAT IS ALREADY KNOWN ON THIS TOPIC**

- Many patient safety practices can reduce risk of harm to patients, but are often not implemented routinely.
- Insights from implementation science may help address this challenge.

**WHAT THIS STUDY ADDS**

- This review provides an overview of the strategies and factors reported to influence implementation of three commonly used safety practices: rapid response systems, antibiotic stewardship and medication reconciliation.
- The included studies lacked active mapping of implementation factors and continuous adaptation of practices and activities to these.

**HOW THIS STUDY MIGHT AFFECT RESEARCH, PRACTICE OR POLICY**

- Practitioners may benefit from our findings to inform themselves of current implementation practice as well as potential context factors to be aware of.
- Future research could inform implementation practice by exploring the relation between specific implementation activities and the factors they aim to address.

## Introduction

 Patient safety practices have been developed to reduce risk of excess morbidity and mortality for a variety of patient groups and health services. Many such practices are shown to have the potential of saving lives and reducing the risk of errors when successfully implemented.^1^,^3^ Yet, effectively integrating them into routine clinical practice, in a scalable and sustainable manner, can be challenging.^4 5^ Implementation science^6 7^ in addition to theories and methods from improvement science,^8 9^ can help address these challenges of implementation.^10^,^12^

The field of patient safety has traditionally been grounded in quality improvement and improvement science, the latter aspiring to produce generalisable knowledge on how to improve quality of care.^8 11^ Stepwise, iterative methods, such as the Model for Improvement,^8^ are often used to guide implementation of patient safety practices, and the cyclical Plan-Do-Study-Act (PDSA) model for small-scale testing of interventions is used widely.^8^ Sharing the goal of improving care, but originating in the evidence-based medicine movement, implementation science aims to promote the systematic uptake of research findings into routine practice.^6^ Influenced by behavioural and social sciences, it often takes more theory-driven approaches as it seeks to understand and explain the determinants of implementation success.^11^

A large number of implementation science theories, models and frameworks (TMFs) have been described, but evidence is limited on how to select among these, and their respective effects when applied.^12^,^14^ In a proposed taxonomy, Nilsen describes three overarching aims: describing/guiding the process of implementation, explaining what may influence the implementation and evaluating the implementation.^15^ Across TMFs, it is thus possible to characterise implementation by: (1) The *implementation activities* used, including strategies and actions to enable implementation and sustainment of a practice.^16 17^ (2) The *implementation factors*, referring to elements of the context surrounding the implementation, expected to affect the implementation positively or negatively and often termed facilitators or barriers.^18^,^21^ (3) *Implementation outcomes*, indicators of implementation success, such as fidelity or sustainability. These outcomes are distinct from patient outcomes, yet essential to improve them, through understanding and addressing challenges to implementation.^22 23^ Ideally, development of implementation activities should be guided by knowledge about factors in the given context.^24^ This entails a need for repeated measuring of implementation factors and their development.^20 25^

Many patient safety practices are complex interventions,^26^ applied across a wide range of healthcare settings such as hospitals, general practices, nursing homes and mental healthcare services.^27 28^ Given the similarities in the challenges faced when integrating them into everyday practice, there seems to be an opportunity for cross-cutting learning between settings and practices. However, most readily available implementation studies focus on one practice or setting, often in the form of implementation case studies.

The aim of the present study is to better understand the success and failure of implementing patient safety practices, to enable better and more systematic implementation. We studied three practices (see box 1), chosen to represent well-described, internationally used practices that are applied across different health services and patient categories. We sought to answer the following questions, for the implementation of antibiotic stewardship programmes (ASP), medication reconciliation (MedRec) and rapid response systems (RRS):

- Which implementation activities and implementation factors are reported in the existing literature?
- What is the rationale for the activities, and how are activities and factors measured?
- Which combinations of factors and activities were reported to be more or less successful, and can this evidence guide future implementation and research?

Box 1 **Descriptions of the three patient safety practices included in this review**

**Antibiotic stewardship programmes (ASP)** aim to measure and improve how antibiotics are prescribed by clinicians and used by patients. The programmes offer a set of key principles to guide efforts to improve antibiotic use and, therefore, advance patient safety and improve outcomes.^57^ ASPs can increase cure rates while reducing treatment failures, adverse effects, antibiotic resistance, hospital costs and length of hospital stay. Substantial barriers to implement ASP are demonstrated even in hospitals in developed countries.^58^

**Medication reconciliation (MedRec)** aims to ensure accurate and complete medication information at interfaces of care, to avoid adverse drug events.^59^ It is the systematic process of identifying an accurate list of a patient’s current medicines and comparing them with the current list in use, recognising any discrepancies, and documenting any changes, thereby resulting in a complete list of medicines, accurately communicated.^60^ Evidence supports reduction of medication discrepancy rates from MedRec interventions when implementation is systematically supported,^61 62^ but difficulties implementing have been described.^63^

**Rapid response systems (RRS)** aim to identify and respond to patients in clinical deterioration. They have been defined as ‘a whole system for providing a safety net for patients who suddenly become critically ill and have a mismatch of needs and resources’.^64^ RRSs by (international) definition contain four limbs: an afferent limb for identification of the deteriorating patient (sometimes using an early warning score), an efferent limb ensuring relevant care and/or transfer, as well as limbs for process improvement and governance.^64 65^ Evidence supports that RRSs reduce hospital mortality and cardiac arrests.^66^ However, challenges to fidelity and sustainability have been reported.^67^,^69^

## Methods

A scoping review methodology was chosen because of its appropriateness for examining emerging evidence and identifying key characteristics or factors related to a concept.^29^ We sought to identify empirical research reporting implementation of the selected patient safety practices, and discover the implementation activities used as well as implementation factors and implementation outcomes reported. Our method followed established guidelines.^30^,^33^ We performed thematic coding and additional, exploratory analyses (detailed below), to synthesise and convey our data to the reader. Reporting was guided by the Preferred Reporting Items for Systematic Review and Meta-Analyses extension for scoping reviews (PRISMA-ScR).^34^ A protocol was publicly registered.^35^ The review was planned and conducted by a research group including expertise within all three patient safety practices, as well as improvement and implementation sciences theories and methods. Three teams of two researchers worked in parallel for the literature screening, in close collaboration and with frequent and extensive consensus sessions throughout the process.

### Eligibility criteria, information sources and search

The databases searched were Medline, Embase (Excerpt Medica Database), Web of Science, Cumulative Index to Nursing and Allied Health Literature (CINAHL), PsycINFO and Education Resources Information Center (ERIC). Search terms were developed from pilot searches and prior knowledge of the field, and through a consensus process to identify keywords from existing literature. In collaboration with a research librarian, specific terms for each practice were combined with a common search string covering terms related to implementation science, quality improvement and implementation outcomes. To avoid limiting the search we did not include terms regarding specific implementation activities or factors. In addition to free-text word searches we included relevant subject headings in Medline, Embase and CINAHL. As pilot searches showed that relevant studies were mostly published during the last decade, and due to resource constraints, searches were limited to publication year 2011 to present (latest search performed on 16 March 2023). Original research studies or quality improvement reports reporting on any of the three practices were included if information on all three of implementation strategies, implementation factors and implementation outcomes were included, and the report was written in English. Otherwise, all study designs were eligible. The full search strategy and eligibility criteria are included as onlinesupplemental materials 1  2.

### Selection of sources of evidence

To ensure uniform application of methods between the three reviewing teams, our research librarian extracted a purposive calibration sample of 30 papers for each practice (see online supplemental material 3). Abstracts and full texts were then evaluated against our eligibility criteria within and between teams using the Rayyan software platform.^36^ Any discrepancies were discussed and resolved in consensus meetings. It was clear from this initial calibration that implementation activities, factors and outcomes were not always described in the abstract even when present in the full text. Their absence was, therefore, not applied as an exclusion criterion in the abstract screening phase. To increase efficiency of the review, initial screening for eligibility using title and abstract was assisted by ASReview^37^ (V.0.19–1.1), an open-source, active-learning-aided software tool. This ‘researcher-in-the-loop’ artificial intelligence (AI) tool works by continually reprioritising the search results, based on text analysis and using prior decisions by the reviewers. The AI model used recommended standard parameters, and we predefined the stopping criteria as 25% of the total number of unique search results or 100 consecutive irrelevant papers. As a quality control measure, one team (examining the RRS practice) continued the screening to 40%, which did not result in any new inclusions. Two researchers performed the screening independently for each practice. Combining the screening results from both researchers in each team, we further assessed eligible papers based on full texts. In case of disagreement within a team regarding eligibility, consensus was sought in the full research group. If perceived as potentially relevant, references from included papers could be screened for eligibility. Where it became clear during full-text screening that one project or study was reported across several papers, eligibility criteria were applied for the study as a whole. We also assessed any systematic reviews identified during the literature search, and screened the papers included in relevant reviews for eligibility.

### Data charting, synthesis and supplementary analysis

Descriptive information on each publication was collated in a table format with publication year, study design and key implementation outcomes. We further conducted inductive, thematic analysis, to reflect the concrete nature of the patient safety literature and how it explains implementation efforts. An inductive approach was chosen because of our concerns on potential limitations in the existing literature with regard to incomplete reporting and inconsistent use of implementation terminology. Reflecting our research questions and inspired by Nilsens’ description of the three aims of implementation TMFs,^15^ text extracts containing relevant information on implementation activities, factors and outcomes were first sorted to these three overarching categories. Next, the approach was iterative, applying elements of systematic text condensation.^38^ Text extracts were further explored and condensed into thematic categories using NVivo software (V.20.5.2; QSR international). These were compared across practices and processed to reach consensus among the researchers on a set of categories (illustration of the process can be found in online supplemental material 4). Representation of all practices in all categories was not an a priori requirement. For each category, a title, a description and examples were developed. All text extracts were subsequently coded to these categories. Coding was reviewed by a second researcher for a minimum of five publications on each practice to check for consistency of application. Any need for clarification or specification of coding was resolved in consensus meetings. For each included study, the presence of text extracts relating to each category (as a dichotomous variable ‘present’/‘not present’) was entered in SPSS statistics (V.29; IBM), allowing for analysis of frequency and plots of activities, factors and outcomes. Further, as an exploratory step to map the most common combinations of factors and activities, of which the total number of possible specific combinations would be very large, dendrograms based on hierarchical cluster analysis using simple matching coefficient were performed using R statistics.^39^ Wherever it was evident that one intervention/implementation process was reported across several papers, these were analysed as a single case.

## Results

**Figure 1:**
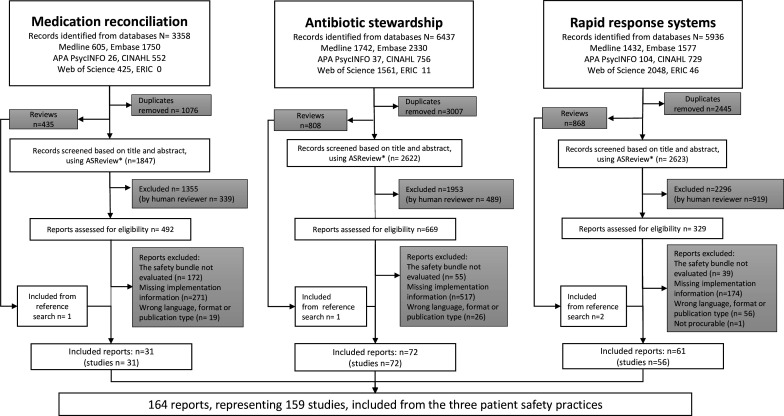
Preferred Reporting Items for Systematic Review and Meta-Analyses (PRISMA) 2020 flow diagram for included papers on medication reconciliation, antibiotic stewardship and rapid response systems. *For description of ASReview methodology, see ‘methods’ section, ‘selection of sources of evidence’. APA, American Psychological Association; ERIC, Education Resources Information Center; CINAHL, Cumulative Index to Nursing and Allied Health Literature.

We included 159 studies: 31 on MedRec, 72 on ASP and 56 on RRS (figure 1, PRISMA flow chart). Most studies were conducted in the USA and within a hospital setting. Even though elements of the RRS practice are relevant across healthcare services, only studies of ASP and MedRec implementation were found in other settings such as nursing homes, general practice and mental health services. We noted a general increase in number of relevant publications over the years with two-thirds of the ASP studies published in 2020 or later. An overview of the included studies and their key findings is provided in online supplemental material 5. The thematic categories resulting from processing the reported implementation activities, implementation factors and implementation outcomes are presented in table 1, with the frequency of reporting for all categories and practices described in table 2.

**Table 1:** Thematic categories of implementation activities, factors and outcomes

Category title	Description	Examples
Implementation activities (actions and strategies to enable implementation and sustainment of a new practice)		
Education	Educational activities using passive or didactic learning.	Lectures on the practice or related subjects. Presentations given as part of introductory programme for new staff. E-learning activities concerning the practice.
Planning and preparation	Preparatory steps to enable compliance to the practice and planning the implementation process.	Consulting experts or stakeholders. Mapping current work processes or existing knowledge about the given practice. Audits to detect current improvement potential. Performing piloting.
Method-based approach	Using defined, systematic methods or guidance from implementation or quality improvement literature.	Applying variations of Plan-Do-Study-Act circles. Establishing dedicated implementation teams. Applying theoretical frameworks. Applying ‘Core Elements’ of antibiotic stewardship.
Audit and feedback	Collecting data that enables evaluation of the implementation, and the use of these for feedback and reflection.	Regular feedback on antibiotic prescription rates, number of medication errors detected, RRT activation or error rates. Audit with feedback to individual clinicians or organisational units.
Motivate and remind	Actions to motivate, support, persuade or remind users.	Ensuring leadership support. Sharing positive stories of practice use. Using champions. Nudging. Distribution of guidelines and pocket cards. Visual or digital reminders. Stakeholder engagement events. Financial incentives.
Resource allocation	Allocation of human, financial or other resources. Technical or organisational changes to facilitate implementation.	Establishing new positions for the practice (pharmacist, RRT nurse). Changing electronic health records to facilitate MedRec or to integrate an EWS. Provision of IT or medical equipment. Establishing committee for continuous oversight (RRS).
Simulation and training	Simulation, interactive team training and practical trainings.	Simulation-based training of rapid response teams. Conducting MedRec under the guidance of an expert. Bedside or interactive, practical training related to the practice.
Patient involvement	Engaging patients or their representatives in implementation processes.	Patients or representatives consulted as stakeholders, as an active part of the new practice or as receivers of care; engaged in development of information targeted at patients or relatives. RRS activation by patients or relatives.
Implementation factors (contextual elements expected to affect implementation positively or negatively)		
Clinical competence and collaboration	Access to relevant clinical and technical knowledge and skills, and multiprofessional collaboration.	Promoting multidisciplinary collaboration and teamwork was important for success. Dedicated RRS nurse to assist with new or infrequent skills was important. Involving pharmacist’s’ expertise was necessary for MedRec. Poor interprofessional collaboration was barrier to activating RRS.
Resources	Availability of necessary time, structures and personnel.	Allocation of dedicated nurse positions facilitated implementation. High staff turnover described as major challenge. Adequate resourcing of RRS was crucial. Implementation was hindered by lack of protocols or equipment. Reducing rework helped the implementation.
Readiness and engagement	Personnel, as individuals and team, feeling inclined and primed to implement the intervention. Personnel engaging in the implementation process.	Improved staff ‘buy-in’ was positive for implementation. Success was driven by motivated and engaged team members. Conservative culture with staff resistant to change was a barrier. Nurse ownership of the RRT resulted in high activation rates.
External influence	Recommendation, demand or support to implement the intervention from outside the organisation (hospital, health trust or equivalent).	Recommendation from national medical association or demand from national health authorities to implement. Implementation required for hospital accreditation. Practice recommended as ‘best practice’.
Organisational involvement	Commitment, support and facilitation of implementation from within the organisation (hospital, health trust or equivalent).	‘Institutional commitment’, ‘organisational sponsorship’ or ‘buy-in from leadership’ stated as important for success of implementation. Lack of organisational support and prioritisation found to be an implementation barrier.
QI competence	Access to and employment of quality improvement knowledge, methods and skills.	Defined improvement/implementation team accelerated implementation. Lack of specialised QI skills hindered implementation. Use of improvement methodology seen as instrumental for sustainment.
Feasibility of intervention	The patient safety intervention is feasible to implement within the context and with the provided resources.	Intervention failed due to excessive workload and alert fatigue. Implementation facilitated by intervention matching existing workflow or workplace culture. Intervention fit to already implemented medication review seen as important.
Implementation outcomes (indicators of various aspects of implementation success)		
Fidelity or compliance	The degree to which the practice, or parts of the practice, is implemented as intended.	Adherence to guidelines or protocol elements. Rate of RRT activations or response times. Percentage of patients with reconciled medication list within 24 hours of admission. Incomplete or wrong EWS.
Proxy indicator for fidelity	Parameters expected to be a consequence of implementation, but not a direct measurement of it.	Antibiotic usage rate. Cardiac arrest or ICU transfer rate. Number of unintentional discrepancies between medication lists.
Sustainability	Duration of achieved implementation outcome. Often defined by study authors as sustainability measures.	Repeat measurements of implementation outcomes postimplementation.
Acceptability	Users’ satisfaction with and acceptance of the new practice.	Users’ satisfaction with the practice. User’s perception of the practice as clinically meaningful. Feasibility when stated as outcome by authors. Assessed by survey or interview.
Spread	Extension of the new practice within the organisation (hospital, health trust or equivalent).	Successful implementation in one ward led to implementation across entire facility. Initial implementation led to deployment of RRS in the entire hospital group.

Examples intend to be typical of the category, are non-exhaustive and may be specific to one practice or summarised for several practices.

EWS, Early Warning Score; ICU, intensive care unit; IT, information technology; MedRec, medication reconciliation; QI, Quality Improvement; RRS, rapid response system; RRT, rapid response team.

**Table 2:** Absolute and relative frequency of studies reporting one or more implementation activity, factor or outcome from each thematic category

	All practices (n=159)	ASP (n=72)	RRS (n=56)	MedRec (n=31)
Implementation activities				
Education	124 (78%)	50 (69%)	50 (89%)	24 (77%)
Planning and preparation	111 (70%)	34 (47%)	50 (89%)	27 (87%)
Method-based approach	111 (70%)	52 (72%)	32 (57%)	27 (87%)
Audit and feedback	103 (65%)	41 (60%)	32 (57%)	27 (87%)
Motivate and remind	80 (50%)	26 (36%)	31 (55%)	23 (74%)
Resource allocation	77 (48%)	17 (24%)	39 (70%)	21 (68%)
Simulation and training	32 (20%)	6 (8%)	21 (38%)	5 (16%)
Patient involvement	18 (11%)	8 (11%)	5 (9%)	6 (16%)
Implementation factors				
Clinical competence and collaboration	101 (64%)	42 (58%)	36 (64%)	23 (72%)
Resources	74 (47%)	21 (29%)	33 (59%)	20 (65%)
Readiness and engagement	69 (43%)	15 (21%)	35 (63%)	19 (61%)
External influences	64 (40%)	29 (40%)	18 (32%)	17 (55%)
Organisational involvement	58 (37%)	17 (24%)	31 (55%)	10 (32%)
QI competence	55 (35%)	26 (36%)	13 (23%)	16 (52%)
Feasibility of intervention	37 (23%)	11 (15%)	14 (25%)	12 (39%)
Implementation outcomes				
Fidelity or compliance	117 (74%)	39 (54%)	52 (93%)	26 (84%)
Proxy indicator for fidelity	90 (57%)	48 (67%)	25 (45%)	17 (55%)
Sustainability	77 (48%)	44 (61%)	21 (38%)	12 (39%)
Acceptability	37 (23%)	8 (11%)	25 (45%)	4 (13%
Spread	18 (11%)	9 (13%)	4 (7%)	5 (16%)

ASP, antibiotic stewardship programmes; MedRec, medication reconciliation; RRS, rapid response system.

The implementation activities, factors and outcomes showed common traits across the three reviewed practices, countries of implementation and health services. These are reviewed in more detail in the sections that follow.

### Implementation activities

We identified a wide range of activities used, which we grouped into eight thematic categories. The most frequently reported were: *education*, *planning and preparation*, *method-based approach*, and *audit and feedback*. These categories were reported by at least two-thirds of the studies. The categories *motivate and remind* and *resource allocation* were found in approximately half of the studies, while less commonly reported activities were from the *simulation and training* and *patient involvement* categories. Most studies reported activities from multiple categories, with a median of four categories reported per study (IQR=2). Studies on ASP implementation reported fewer activities (median=3, IQR=2) than RRS and MedRec (both median=5, IQR=2). All categories of activities were represented in each of the three safety practices. Across practices, activities from the *motivate and remind* and *method-based approach* categories were reported most often by MedRec studies, while *education* and *simulation and training* were reported most often in the RRS studies. *Planning and preparation*, *resource allocation* and *motivate and remind* were reported more rarely by ASP studies, compared with the two other practices. Explicit rationale for or measurements on the enactment or completion of the activities were rarely reported. Examples of quantified activities reported in the studies included numbers of staff trained or number of PDSA cycles completed.

### Implementation factors

The reporting of factors relevant to the implementation outcomes was less detailed than descriptions of the activities. We identified seven thematic categories of factors. The most to least frequently reported were: c*linical competence and collaboration, resources, readiness and engagement, external influence, organisational involvement, QI competence,* and *feasibility of innovation*. Most papers (n=89, 56%) reported factors from three or more of the seven categories (median=3, IQR=2). Studies from the ASP literature tended to report fewer factors (median=2, IQR=2) than RRS (median=3, IQR=2) and MedRec (median=4, IQR=2). Some differences were noted in how frequently the factors were reported across the practices: The *feasibility of intervention* category was reported most often in the MedRec studies, and o*rganisational involvement* reported most often in the RRS studies. The ASP studies reported least often on *resources* and *readiness and engagement*. Most of the reported factors were not based on prospectively collected implementation-related metrics but based on the authors’ opinion or retrospectively performed surveys or interviews. Very few studies provided data detailing the suggested factors’ impact on the implementation process. One such study, by Yadav *et al*, describes a thorough preimplementation mapping and analysis of stakeholders’ perceptions and local characteristics, followed by a dynamic process using feedback to continuously adapt the implementation support.^40^

### Implementation outcomes and success

We identified five thematic categories of implementation outcomes. The most to least frequently reported were: *fidelity or compliance, proxy indicator for fidelity, sustainability, acceptability* and *spread*. Across practices, the *acceptability* category was reported most often by RRS studies, and the *sustainability* category most often by ASP studies. The *fidelity or compliance* category was reported least often by ASP studies. However, ASP studies reported more often on outcomes from the *proxy indicator for fidelity* category, such as antibiotic usage rate. Several variations of outcomes were found within each category. Predefined criteria for successful implementation were rarely stated in the studies. Comparing outcomes to recognised standards or external demands was also rarely reported. Despite this, a majority of studies reported some degree of successful implementation, either directly stated or implied. Also, some studies reported on previous or initial failure, followed by successful re-implementation or improvement. Only 3 of the 159 studies explicitly reported failed implementation.^41^,^43^

### Combinations of activities and factors

**Figure 2:**
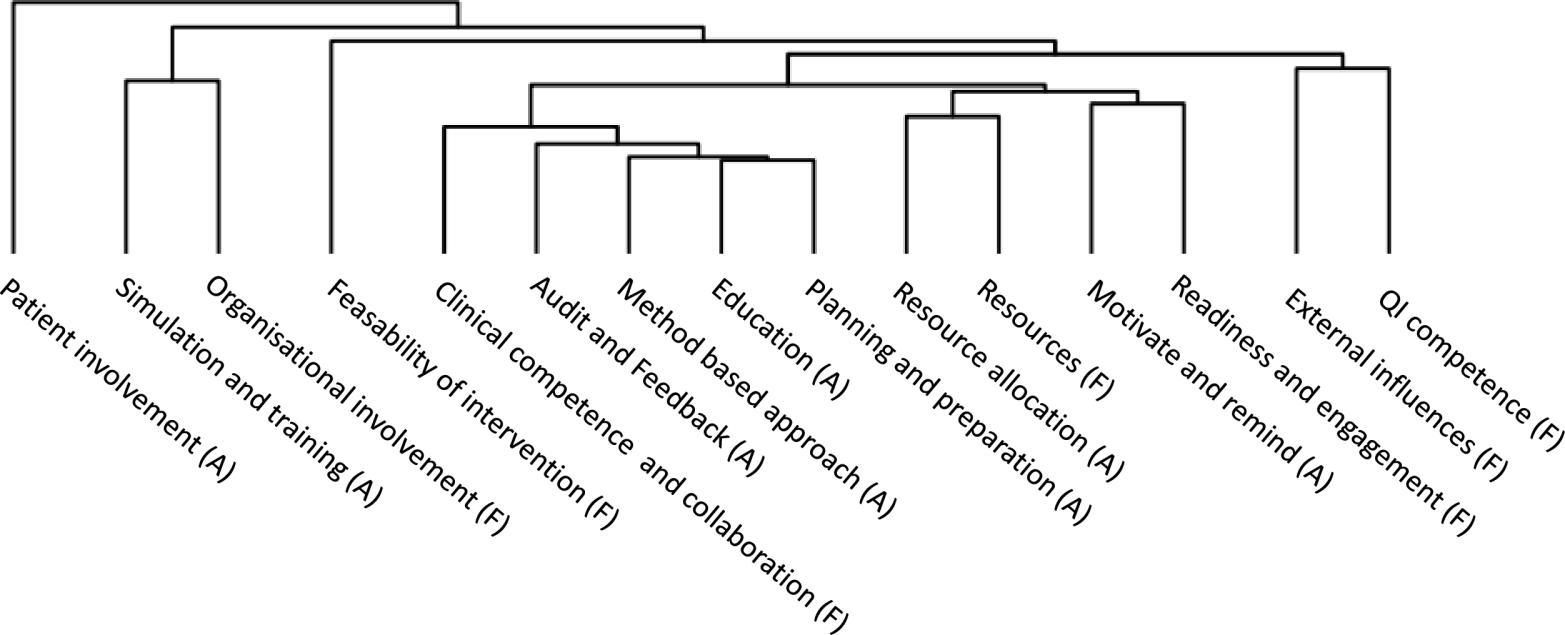
*:* Dendrogram of hierarchical cluster analysis of implementation activities and implementation factors for all practices, using simple matching coefficient. Clustering is indicated by the horizontal lines, and vertical distance indicate degree of difference.

A variety of combinations of implementation activities and factors were reported in the included studies. The hierarchical cluster analysis presented as a dendrogram in figure 2 suggested two more common combinations. The first combination includes the activities *planning and preparation*, *method-based approach*, *education*, *audit and feedback*, and the factor *clinical competence and collaboration*. Thirty-six studies (23%) reported this exact combination, while additional 47 (30%) studies reported four out of five of these activities and factors. The second combination includes the activities *motivate and remind* and *resource allocation* and the factors *readiness and engagement* and *resources*. Twenty studies (13%) reported this combination, while additional 34 (21%) reported three out of four of these activities and factors. Cross-tabulations of combinations of activities or factors support this positive clustering. The two clusters did not appear mutually exclusive, as many studies reported on elements from both clusters.

## Discussion

This scoping review of the literature on implementation of three established patient safety practices reveals a close-to-practice description of the range of activities used to enable implementation and the implementation factors suggested to affect it. A trend towards combining certain activities and factors was found, yet the rationale for the chosen activities was often poorly described.

### Implementation activities

The great variety of implementation activities reported corresponds well with the multitude of activities found in relevant implementation science compilations such as the Expert Recommendations for Implementing Change (ERIC) taxonomy that identifies 73 distinct strategies.^17^ Many of the activities reported seem to stem directly from improvement science, such as those in our category *method-based approach*, in which a majority of activities are highly influenced by Deming’s PDSA cycle.^8^ Likewise, activities from the categories *education* and a*udit and feedback*, are recognisable from the *Model for Improvement*.^8^ These three categories were included in the most frequently used combinations of activities suggested by cluster analysis (figure 2, first combination). Comparing with the Consolidated Framework for implementation Research (CFIR),^20^ our activity categories relate to the *implementation process* domain, which also seem grounded in the PDSA model. The combination of multiple activities in each study may relate to both improvement and implementation science recommendations on so-called intervention ‘bundles’^44 45^ or ‘combined strategies’.^20^ How activities were selected and their rationale or function was, however, often not reported in the included literature. This leaves it challenging to systematically apply taxonomies such as ERIC. We note that while *audit and provide feedback* is one discrete ERIC strategy, we identified this as a comparable thematic category encompassing several activities. Likewise, our category of *simulation and training* seems to correspond to the single ERIC strategy of *make training dynamic* but could also be related to others such as *promote adaptability* or *model and simulate change*. The sparse reporting hinders both the possibility of interpreting our findings to existing TMFs, and our aim to investigate the rationale for and measurement of implementation activities. Such a gap in explaining activities and their relation to implementation outcomes has been previously described, and formulating a theory on *how* and *why* an intervention may work is recommended to better understand it.^46^

### Implementation factors

The reported implementation factors were most often suggested in retrospect, often as an expert opinion, and were rarely measured. Their actual importance for implementation, or whether they were directly aligned with the selected implementation activities (or impacted by them), thus remains unclear. Notwithstanding this limitation, most factors reported in the included literature could relate to several established implementation science frameworks such as Model for Understanding Success in Quality,^47^ Promoting Action on Research Implementation in Health Services^48^ or CFIR.^20^ Relating specifically to CFIR, we note commonalities between our category *external influence* and the CFIR domain *outer setting, a*nd our category *feasibility of intervention* to the CFIR *innovation* domain. As our thematic categories reflect what is reported in this specific body of literature, the prominence of *QI competence* and *clinical competence and collaboration as* separate categories could signal a QI influence, but also reflect the nature of the patient safety practices.

### Implementation outcomes and success

Many studies lacked predefined criteria for success or comparison of outcomes to established standards or outcomes that adequately evaluated essential aspects of the practice. Relating the reported implementation actions and factors to more or less successful implementation thus proved infeasible, illustrating the nature of the included literature and the complexity of defining ‘success’. While most outcomes reported were relatable to the taxonomy proposed by Proctor *et al*,^22^ one of the most used outcome categories revealed by our inductive method, *proxy indicator for fidelity,* could illustrate the need for additional outcome measures beyond those suggested. Typical examples were number of medication list errors detected by MedRec and antibiotic usage rate, both indicating patient safety risk at system level. Most studies reported successful implementation, which does not align with the levels of implementation failure generally reported in the literature.^4 5^ We suspect that part of the explanation is bias, both through selective publication of successful implementations, and through increased chances of success when implementations are reported as research projects entailing larger availability of resources and expertise.

### The complex relations between implementation activities, factors and outcomes

System-oriented implementation science literature sees activities, factors and outcomes as interacting and evolving, highlighting continuous optimisation of the activities to fit the implementation context.^49 50^ However, many studies included in this review seem to employ a more linear approach, such as methods inspired by Statistical Process Control^8^ to test the effects of single activities one by one.^51 52^ We found a few notable studies that reported thorough preimplementation assessment of the context and adaptation of the intervention and the implementation activities to ensure staff’s engagement and motivation and address structural hindrances.^40 53 54^ Examining whether the reported activities aligned to the reported factors in the included studies proved infeasible, due to the limitations in the reporting as outlined above. Adaptive system thinking^50^ does not seem to have a prominent role in the examined literature on implementation of patient safety practices.

### Limitations

A risk of missing relevant studies or information is present in our review. We applied a machine learning (ML) methodology (ASReview software^37^) in our initial eligibility screening of titles and abstracts. Although we found this an effective addition to the review methods used, there are no universally accepted standards for stopping criteria for ML-assisted reviews^55^ which entails a risk of missing potential inclusions. We assume this risk to be low, as we observed a clear pattern of prioritising from the software and performed a quality check for one practice where proceeding beyond the stopping criteria did not result in new inclusions. The risk of insufficient recall is also present when screening manually.^56^ Further, we did not include purely qualitative studies on implementation factors if they did not report on implementation outcomes. Although this choice was intended to produce a more coherent and analysable data set for our research questions, we cannot exclude that such studies may contain relevant information. Lastly, some studies were described in several papers with different foci. This model of reporting entails a risk of incomplete descriptions or studies not being included, if all papers are not found and linked during the review process.

## Conclusions and implications for research and practice

The literature on implementation of three patient safety practices, namely antibiotic stewardship programmes, medication reconciliation and rapid response systems, describes a range of activities used and the context factors thought to be relevant for successful implementation.

Practitioners seeking to implement these safety practices may benefit from this summary of key aspects of current implementation practice. The dominant approach uses bundles of activities directed at enabling and engaging clinical staff, through activities to improve clinical and QI competencies, provide feedback and motivate staff. Across practices, the most common context factors relate to knowledge, skills and multiprofessional collaboration. We suggest that future implementations of patient safety practices consider actively mapping barriers and facilitators, to adapt activity bundles accordingly.

Method-based improvement science, and to a lesser degree determinant frameworks from implementation science, seem to dominate the scientific approach in the included literature, with limited focus on complex adaptive systems. We suggest that future studies on the implementation of patient safety practices explore how adaptation of activities to address changing contextual implementation factors impact the implementation outcomes, and that such studies should report the rationale for implementation activities and measurements of their enactment, to better understand the mechanisms behind effective activities. This would be further helped by clearly reported criteria for implementation success. Finally, we could learn from studies of failed implementations, of which few are published. Addressing these issues could prove an untapped potential for improving practice.

## Supplementary material

10.1136/bmjqs-2024-017185 online supplemental material 1

10.1136/bmjqs-2024-017185 online supplemental material 2

10.1136/bmjqs-2024-017185 online supplemental material 3

10.1136/bmjqs-2024-017185 online supplemental material 4

10.1136/bmjqs-2024-017185 online supplemental material 5

## Data Availability

Data are available upon reasonable request.
