# Supplementary material for: Rapid response systems, antibiotic stewardship and medication reconciliation: a scoping review on implementation factors, activities and outcomes
Source: BMJ Qual Saf. 2024 Jun 6;34(4):e017185. doi: 10.1136/bmjqs-2024-017185 (PMC12013571; doi:10.1136/bmjqs-2024-017185)
Supplement: online supplemental material 1 [file bmjqs-34-4-s001.pdf]

SUPPLEMENTARY MATERIAL 1: SEARCH STRATEGY

Antibiotic Stewardship programmes:

Ovid MEDLINE(R) and Epub Ahead of Print, In-Process, In-Data-Review & Other Non-Indexed Citations and Daily <1946 to March 15, 2023> Search Date 16 March 2023

1 implementation science/ 1224

2 Quality Improvement/ 32098

3 Process Assessment, Health Care/ 4981

4 implement*.ti. 72725

5 ((implement* or improv*) adj3 (science* or research* or strateg* or process* or evidence-based or framework* or context or mechanism* or activit* or factor* or success* or succeed* or fail* or effective* or fidelity or utility)).ti,ab,kf. 359686

6 (factor* adj2 (hinder* or barrier* or facilitat* or mediat* or moderat* or promot*)).ti,ab,kf. 55827

7 (Quality adj2 (improv* or management)).ti,ab,kf. 178084

8 (process measure* or process assessment* or process evaluation*).ti,ab,kf. 9010

9 ((complian* or comply or complie* or adhere*) adj2 guideline*).ti,ab,kf. 8237

10 1 or 2 or 3 or 4 or 5 or 6 or 7 or 8 or 9 640054

11 Antimicrobial Stewardship/ 3266

12 (ASP or antimicrobial stewardship program* or anti-microbial stewardship program* or antibiotic stewardship program* or anti-biotic stewardship program* or antibiotic prescribing or antibiotic prescription* or anti-biotic prescribing or anti-biotic prescription*).ti,ab,kf. 42060

13 11 or 12 44005

14 10 and 13 2021

15 limit 14 to yr="2011 -Current" 1742

16 limit 15 to (meta analysis or "review" or "systematic review") 241

17 15 not 16 1501

https://ovidsp.ovid.com/ovidweb.cgi?T=JS&NEWS=N&PAGE=main&SHAREDSEARCHID=lbtnuhtu3Ad4rJ8v0zr9gTZKVbcws7lXHlopLMb5B62g4OwgCgmMFuTBaSBVyKVP

Embase (Ovid) <1974 to 2023 March 15>; Search Date 16 March 2023

1 implementation science/ 4286

2 total quality management/ 89035

3 implement*.ti. 99653

4 ((implement* or improv*) adj3 (science* or research* or strateg* or process* or evidence-based or framework* or context or mechanism* or activit* or factor* or success* or succeed* or fail* or effective* or fidelity or utility)).ti,ab,kf. 481616

5 (factor* adj2 (hinder* or barrier* or facilitat* or mediat* or moderat* or promot*)).ti,ab,kf. 68937

6 (Quality adj2 (improv* or management)).ti,ab,kf. 272538

7 (process measure* or process assessment* or process evaluation*).ti,ab,kf. 12126

8 ((complian* or comply or complie* or adhere*) adj2 guideline*).ti,ab,kf. 14396

9 1 or 2 or 3 or 4 or 5 or 6 or 7 or 8 891438

10 antimicrobial stewardship/ 10401

11 (ASP or antimicrobial stewardship program* or anti-microbial stewardship program* or antibiotic stewardship program* or anti-biotic stewardship program* or antibiotic prescribing or antibiotic prescription* or anti-biotic prescribing or anti-biotic prescription*).ti,ab,kf. 53164

12 10 or 11 59792

13 9 and 12 3701

14 limit 13 to conference abstract 1053

15 13 not 14 2648

16 limit 15 to yr="2011 -Current" 2330

17 limit 16 to "review" 323

18 (meta-analysis or metaanalysis).ti. 217131

19 16 and 18 12

20 17 or 19 331

21 16 not 20 1999

https://ovidsp.ovid.com/ovidweb.cgi?T=JS&NEWS=N&PAGE=main&SHAREDSEARCHID=JHxjujsUdp9dOjxnflFp5VtxGaXf69yE8oOzu3moflFWqFhf5UYR9bGhanytDv0z

APA PsycInfo <1806 to March Week 1 2023> ; Search Date 16 March 2023

1 implement*.ti. 23586

2 ((implement* or improv*) adj3 (science* or research* or strateg* or process* or evidence-based or framework* or context or mechanism* or activit* or factor* or success* or succeed* or fail* or effective* or fidelity or utility)).tw. 98083

3 (factor* adj2 (hinder* or barrier* or facilitat* or mediat* or moderat* or promot*)).tw. 17207

4 (Quality adj2 (improv* or management)).tw. 30346

5 (process measure* or process assessment* or process evaluation*).tw.3576

6 ((complian* or comply or complie* or adhere*) adj2 guideline*).tw. 948

7 1 or 2 or 3 or 4 or 5 or 6 156074

8 (ASP or antimicrobial stewardship program* or anti-microbial stewardship program* or antibiotic stewardship program* or anti-biotic stewardship program* or antibiotic prescribing or antibiotic prescription* or anti-biotic prescribing or anti-biotic prescription*).tw. 1008

9 7 and 868

10 limit 9 to ("0200 book" or "0240 authored book" or "0280 edited book" or "0300 encyclopedia" or "0400 dissertation abstract") 18

11 9 not 10 50

12 limit 11 to yr="2011 -Current" 37

13 limit 12 to "reviews (maximizes specificity)" 5

14 12 not 13 32

https://ovidsp.ovid.com/ovidweb.cgi?T=JS&NEWS=N&PAGE=main&SHAREDSEARCHID=3Dxr78KYiUMKU6Gj7qNy5baI44lLrQwXjhSiXq89SYVHLbLL5hJgcRDwJFwOhN3Jh

CINAHL - EBSCOhost Research Databases, Advanced Search, 1981-current
Search Date 16 March 2023

S1 (MH "Implementation Science") 827

S2 (MH "Quality Improvement") 66,517

S3 (MH "Process Assessment (Health Care)") 4,909

S4 TI implement* 45,429

S5 TI ((implement* or improv*) N2 (science* or research* or strateg* or process* or evidence-based or framework* or context or mechanism* or activit* or factor* or success* or succeed* or fail* or effective* or fidelity or utility)) OR AB ((implement* or improv*) N2 (science* or research* or strateg* or process* or evidence-based or framework* or context or mechanism* or activit* or factor* or success* or succeed* or fail* or effective* or fidelity or utility)) 123,993

S6 TI (factor* N1 (hinder* or barrier* or facilitat* or mediat* or moderat* or promot*)) OR AB (factor* N1 (hinder* or barrier* or facilitat* or mediat* or moderat* or promot*)) 11,819

S7 TI (Quality N1 (improv* or management)) OR AB (Quality N1 (improv* or management)) 76,190

S8 TI ("process measure*" or "process assessment*" or "process evaluation*") OR AB ("process measure*" or "process assessment*" or "process evaluation*") 4,853

S9 TI ((complian* or comply or complie* or adhere*) N1 guideline*) OR AB ((complian* or comply or complie* or adhere*) N1 guideline*)3,548

S10 S1 OR S2 OR S3 OR S4 OR S5 OR S6 OR S7 OR S8 OR S9 276,461

S11 (MH "Antimicrobial Stewardship") 1,556

S12 TI (ASP or "antimicrobial stewardship program*" or "anti-microbial stewardship program*" or "antibiotic stewardship program*" or "anti-biotic stewardship program*" or "antibiotic prescribing" or "antibiotic prescription*" or "anti-biotic prescribing" or "anti-biotic prescription*") OR AB (ASP or "antimicrobial stewardship program*" or "anti-microbial stewardship program*" or "antibiotic stewardship program*" or "anti-biotic stewardship program*" or "antibiotic prescribing" or "antibiotic prescription*" or "anti-biotic prescribing" or "anti-biotic prescription"*) 4,647

S13 S11 OR S12 5,742

S14 S10 AND S13 808

S15 S10 AND S13, Limiters - Published Date: 20110101-20231231 756

S16 Limiters - Publication Type: Meta Analysis, Meta Synthesis, Review, Systematic Review 512,729

S17 S15 AND S16 66

S18 S15 NOT S17 690

Web of Science Core Collection (Clarivate) - (WOS.SCI: 1945 to 2023, WOS.AHCI: 1975 to 2023, WOS.ESCI: 2018 to 2023, WOS.SSCI: 1956 to 2023);
Search Date 16 March 2023

1: TS=(ASP or "antimicrobial stewardship program*" or "anti-microbial stewardship program*" or "antibiotic stewardship program*" or "anti-biotic stewardship program*" or "antibiotic prescribing" or "antibiotic prescription*" or "anti-biotic prescribing" or "anti-biotic prescription*")
Results: 49184

2: TI=(implement*) Results: 167943

3: TS=((implement* or improv*) NEAR/2 (science* or research* or strateg* or process* or evidence-based or framework* or context or mechanism* or activit* or factor* or success* or succeed* or fail* or effective* or fidelity or utility)) Results: 637263

4: TS=(factor* NEAR/1 (hinder* or barrier* or facilitat* or mediat* or moderat* or promot*)) Results: 68328

5: TS=(Quality NEAR/1 (improv* or management)) Results: 249926

6: TS=("process measure*" or "process assessment*" or "process evaluation*") Results: 12759

7: TS=((complian* or comply or complie* or adhere*) NEAR/1 guideline*) Results: 8732

8: (((((#2) OR #3) OR #4) OR #5) OR #6) OR #7 Results: 1062466

9: (#1 AND #8) AND ((PY==("2023" OR "2022" OR "2021" OR "2020" OR "2019" OR "2018" OR "2017" OR "2016" OR "2015" OR "2014" OR "2013" OR "2012" OR "2011")) NOT (DT==("REVIEW" OR "MEETING ABSTRACT" OR "PROCEEDINGS PAPER"))) Results: 1397

10: (#1 AND #8) AND ((PY==("2023" OR "2022" OR "2021" OR "2020" OR "2019" OR "2018" OR "2017" OR "2016" OR "2015" OR "2014" OR "2013" OR "2012" OR "2011")))
Results: 1613

11: (#1 AND #8) AND ((PY==("2023" OR "2022" OR "2021" OR "2020" OR "2019" OR "2018" OR "2017" OR "2016" OR "2015" OR "2014" OR "2013" OR "2012" OR "2011"))) and Review Article (Document Types) Results: 164

Comments: total number of hits = 164 reviews (line 11) + 1397 different types of articles (line 9) (but not reviews, meeting abstracts. There could be editorials, letters or others which are not excluded specifically. Therefore line 10 is exceeds the sum of hits in line 9 and 11.

ERIC (EBSCOhost Research Databases) 1966- current
Search Date 16 March 2023

S1 TI implement* 19,089

S2 TI ((implement* or improv*) N2 (science* or research* or strateg* or process* or evidence-based or framework* or context or mechanism* or activit* or factor* or success* or succeed* or fail* or effective* or fidelity or utility)) OR AB ((implement* or improv*) N2 (science* or research* or strateg* or process* or evidence-based or framework* or context or mechanism* or activit* or factor* or success* or succeed* or fail* or effective* or fidelity or utility)) 57,550

S3 TI (factor* N1 (hinder* or barrier* or facilitat* or mediat* or moderat* or promot*)) OR AB (factor* N1 (hinder* or barrier* or facilitat* or mediat* or moderat* or promot*)) 3,245

S4 TI (Quality N1 (improv* or management)) OR AB (Quality N1 (improv* or management)) 12,679

S5 TI ("process measure*" or "process assessment*" or "process evaluation*") OR AB ("process measure*" or "process assessment*" or "process evaluation*") 1,275

S6 TI ((complian* or comply or complie* or adhere*) N1 guideline*) OR AB ((complian* or comply or complie* or adhere*) N1 guideline*)92

S7 S1 OR S2 OR S3 OR S4 OR S5 OR S6 85,098

S8 TI (ASP or "antimicrobial stewardship program*" or "anti-microbial stewardship program*" or "antibiotic stewardship program*" or "anti-biotic stewardship program*" or "antibiotic prescribing" or "antibiotic prescription*" or "anti-biotic prescribing" or "anti-biotic prescription*") OR AB (ASP or "antimicrobial stewardship program*" or "anti-microbial stewardship program*" or "antibiotic stewardship program*" or "anti-biotic stewardship program*" or "antibiotic prescribing" or "antibiotic prescription*" or "anti-biotic prescribing" or "anti-biotic prescription*") 172

S9 S7 AND S8 19

S10 S7 AND S8,

Limiters - Date Published: 20110101-20231231 11

S11 TI metaanalys* OR meta-analys* or "review" 148,741

S12 S10 AND S11 1

S13 S10 not S12 10

Rapid response systems:

Ovid MEDLINE(R) and Epub Ahead of Print, In-Process, In-Data-Review & Other Non-Indexed Citations and Daily <1946 to March 15, 2023> Search Date 16 March 2023

1 implementation science/ 1224

2 Quality Improvement/ 32098

3 Process Assessment, Health Care/ 4981

4 implement*.ti. 72725

5 ((implement* or improv*) adj3 (science* or research* or strateg* or process* or evidence-based or framework* or context or mechanism* or activit* or factor* or success* or succeed* or fail* or effective* or fidelity or utility)).ti,ab,kf. 359686

6 (factor* adj2 (hinder* or barrier* or facilitat* or mediat* or moderat* or promot*)).ti,ab,kf. 55827

7 (Quality adj2 (improv* or management)).ti,ab,kf. 178084

8 (process measure* or process assessment* or process evaluation*).ti,ab,kf. 9010

9 ((complian* or comply or complie* or adhere*) adj2 guideline*).ti,ab,kf. 8237

10 1 or 2 or 3 or 4 or 5 or 6 or 7 or 8 or 9 640054

11 Hospital Rapid Response Team/ 1004

12 (rapid response system* or RRS or rapid response team* or RRT or medical emergency team* or medical response team* or emergency medical team* or patient-at-risk team* or critical care outreach or intensive care outreach).ti,ab,kf. 21016

13 early warning score/ 372

14 (early warning or "track and trigger" or warning score* or warning system*).ti,ab,kf. 10948

15 (deteriorat* adj3 (early detection or early recognition)).ti,ab,kf. 236

16 11 or 12 or 13 or 14 or 15 32082

17 10 and 16 1676

18 limit 17 to yr="2011 -Current" 1432

19 limit 18 to (meta analysis or "review" or "systematic review") 255

20 18 not 19 1177

https://ovidsp.ovid.com/ovidweb.cgi?T=JS&NEWS=N&PAGE=main&SHAREDSEARCHID=6FzCifdruYSLyJv9BIbTByjiCkrTNARxRt51HMZbzP9YVEMPvjCxwmM4x3B1ta7nw

Embase (Ovid) <1974 to 2023 March 15>; Search Date 16 March 2023

1 implementation science/ 4286

2 total quality management/ 89035

3 implement*.ti. 99653

4 ((implement* or improv*) adj3 (science* or research* or strateg* or process* or evidence-based or framework* or context or mechanism* or activit* or factor* or success* or succeed* or fail* or effective* or fidelity or utility)).ti,ab,kf. 481616

5 (factor* adj2 (hinder* or barrier* or facilitat* or mediat* or moderat* or promot*)).ti,ab,kf. 68937

6 (Quality adj2 (improv* or management)).ti,ab,kf. 272538

7 (process measure* or process assessment* or process evaluation*).ti,ab,kf. 12126

8 ((complian* or comply or complie* or adhere*) adj2 guideline*).ti,ab,kf. 14396

9 1 or 2 or 3 or 4 or 5 or 6 or 7 or 8 891438

10 rapid response team/ 3197

11 (rapid response system* or RRS or rapid response team* or RRT or medical emergency team* or medical response team* or emergency medical team* or patient-at-risk team* or critical care outreach or intensive care outreach).ti,ab,kf. 31178

12 exp early warning score/ 1704

13 (early warning or "track and trigger" or warning score* or warning system*).ti,ab,kf. 14111

14 (deteriorat* adj3 (early detection or early recognition)).ti,ab,kf. 378

15 10 or 11 or 12 or 13 or 14 46280

16 9 and 15 2702

17 limit 16 to conference abstract 835

18 16 not 17 1867

19 limit 18 to yr="2011 -Current" 1577

20 limit 19 to "review" 213

21 (meta-analysis or metaanalysis).ti. 217131

22 19 and 21 67

23 20 or 22 253

24 19 not 23 1324

https://ovidsp.ovid.com/ovidweb.cgi?T=JS&NEWS=N&PAGE=main&SHAREDSEARCHID=2zkIfR26KlrRq0GyJlCtiLYLhBtiBHJsHsSNVJlnNSAg20SAgBcVsHK0LXlvymE3m

APA PsycInfo (Ovid) <1806 to March Week 1 2023> ; Search Date 16 March 2023

1 implement*.ti. 23586

2 ((implement* or improv*) adj3 (science* or research* or strateg* or process* or evidence-based or framework* or context or mechanism* or activit* or factor* or success* or succeed* or fail* or effective* or fidelity or utility)).tw. 98083

3 (factor* adj2 (hinder* or barrier* or facilitat* or mediat* or moderat* or promot*)).tw. 17207

4 (Quality adj2 (improv* or management)).tw. 30346

5 (process measure* or process assessment* or process evaluation*).tw.3576

6 ((complian* or comply or complie* or adhere*) adj2 guideline*).tw. 948

7 1 or 2 or 3 or 4 or 5 or 6 156074

8 (rapid response system* or RRS or rapid response team* or RRT or medical emergency team* or medical response team* or emergency medical team* or patient-at-risk team* or critical care outreach or intensive care outreach).tw. 1319

9 (early warning or "track and trigger" or warning score* or warning system*).tw. 1385

10 (deteriorat* adj3 (early detection or early recognition)).tw. 25

11 8 or 9 or 10 2691

12 7 and 11 168

13 limit 12 to ("0200 book" or "0240 authored book" or "0280 edited book" or "0300 encyclopedia" or "0400 dissertation abstract") 35

14 12 not 13 133

15 limit 14 to yr="2011 -Current" 104

16 limit 15 to "reviews (maximizes specificity)" 9

17 15 not 16 95

https://ovidsp.ovid.com/ovidweb.cgi?T=JS&NEWS=N&PAGE=main&SHAREDSEARCHID=4IP8z5ZtVcQ0v1vAVRQZcgJ7DW5VYbKazLm23XunUVzJIRSW1tkR5OufYl7FUJV4j

CINAHL (EBSCOhost Research Databases), 1981-current
Search Date 16 March 2023

S1 (MH "Implementation Science") 827

S2 (MH "Quality Improvement") 66,517

S3 (MH "Process Assessment (Health Care)") 4,909

S4 TI implement* 45,429

S5 TI ((implement* or improv*) N2 (science* or research* or strateg* or process* or evidence-based or framework* or context or mechanism* or activit* or factor* or success* or succeed* or fail* or effective* or fidelity or utility)) OR AB ((implement* or improv*) N2 (science* or research* or strateg* or process* or evidence-based or framework* or context or mechanism* or activit* or factor* or success* or succeed* or fail* or effective* or fidelity or utility)) 123,993

S6 TI (factor* N1 (hinder* or barrier* or facilitat* or mediat* or moderat* or promot*)) OR AB (factor* N1 (hinder* or barrier* or facilitat* or mediat* or moderat* or promot*)) 11,819

S7 TI (Quality N1 (improv* or management)) OR AB (Quality N1 (improv* or management)) 76,190

S8 TI ("process measure*" or "process assessment*" or "process evaluation*") OR AB ("process measure*" or "process assessment*" or "process evaluation*") 4,853

S9 TI ((complian* or comply or complie* or adhere*) N1 guideline*) OR AB ((complian* or comply or complie* or adhere*) N1 guideline*)3,548

S10 S1 OR S2 OR S3 OR S4 OR S5 OR S6 OR S7 OR S8 OR S9 276,461

S11 (MH "Rapid Response Team") 439

S12 TI ("rapid response system*" or RRS or "rapid response team*" or RRT or "medical emergency team*" or "medical response team*" or "emergency medical team*" or "patient-at-risk team*" or "critical care outreach" or "intensive care outreach") OR AB ("rapid response system*" or RRS or "rapid response team*" or RRT or "medical emergency team*" or "medical response team*" or "emergency medical team*" or "patient-at-risk team*" or "critical care outreach" or "intensive care outreach") 7,458

S13 (MH "Early Warning Score") 161

S14 TI ("early warning" or "track and trigger" or "warning score*" or "warning system*") OR AB ("early warning" or "track and trigger" or "warning score*" or "warning system*") 3,113

S15 TI (deteriorat* N2 ("early detection" or "early recognition")) OR AB (deteriorat* N2 ("early detection" or "early recognition")) 122

S16 S11 OR S12 OR S13 OR S14 OR S15 10,692

S17 S10 AND S16 887

S18 S10 AND S16,

Limiters - Published Date: 20110101-20231231 729

S19 Limiters - Publication Type: Meta Analysis, Meta Synthesis, Review, Systematic Review 512,729

S20 S18 AND S19 94

S21 S18 NOT S20 635

Web of Science Search Strategy (v0.1)(Clarivate.

Web of Science Core Collection (WOS.SCI: 1945 to 2023, WOS.AHCI: 1975 to 2023, WOS.ESCI: 2018 to 2023, WOS.SSCI: 1956 to 2023)
Search Date 16 March 2023

1: TI=(implement*) Results: 167943

2: TS=((implement* or improv*) NEAR/2 (science* or research* or strateg* or process* or evidence-based or framework* or context or mechanism* or activit* or factor* or success* or succeed* or fail* or effective* or fidelity or utility)) Results: 637263

3: TS=(factor* NEAR/1 (hinder* or barrier* or facilitat* or mediat* or moderat* or promot*)) Results: 68328

4: TS=(Quality NEAR/1 (improv* or management)) Results: 249926

5: TS=("process measure*" or "process assessment*" or "process evaluation*") Results: 12759

6: TS=((complian* or comply or complie* or adhere*) NEAR/1 guideline*) Results: 8732

7: (((((#1) OR #2) OR #3) OR #4) OR #5) OR #6 Results: 1062466

8: TS=("rapid response system*" or RRS or "rapid response team*" or RRT or "medical emergency team*" or "medical response team*" or "emergency medical team*" or "patient-at-risk team*" or "critical care outreach" or "intensive care outreach") Results: 22925

9: TS=("early warning" or "track and trigger" or "warning score*" or "warning system*") Results: 25665

10: TS=(deteriorat* NEAR/2 ("early detection" or "early recognition"))
Results: 221

11: ((#8) OR #9) OR #10 Results: 48213

12: (((#7) AND #11) AND ((PY==("2023" OR "2022" OR "2021" OR "2020" OR "2019" OR "2018" OR "2017" OR "2016" OR "2015" OR "2014" OR "2013" OR "2012" OR "2011")))) NOT (DT==("REVIEW" OR "MEETING ABSTRACT" OR "PROCEEDINGS PAPER" OR "BOOK REVIEW" OR "BOOK CHAPTER"))
Results: 1793

13: (((#7) AND #11) AND ((PY==("2023" OR "2022" OR "2021" OR "2020" OR "2019" OR "2018" OR "2017" OR "2016" OR "2015" OR "2014" OR "2013" OR "2012" OR "2011"))))
Results: 2168

14: (((#7) AND #11) AND ((PY==("2023" OR "2022" OR "2021" OR "2020" OR "2019" OR "2018" OR "2017" OR "2016" OR "2015" OR "2014" OR "2013" OR "2012" OR "2011")))) and Review Article (Document Types) Results: 255

Comments: total number of hits = 255 reviews (line 14) + 1793 different types of articles (but not reviews, meeting abstracts, line 12). There could be editorials, letters or others which are not excluded specifically. Therefore also line 13 exceeds the sum of line 12 and 14.

ERIC (EBSCOhost Research Databases) 1966 to current

Search Date 16 March 2023

S1 TI implement* 19,089

S2 TI ((implement* or improv*) N2 (science* or research* or strateg* or process* or evidence-based or framework* or context or mechanism* or activit* or factor* or success* or succeed* or fail* or effective* or fidelity or utility)) OR AB ((implement* or improv*) N2 (science* or research* or strateg* or process* or evidence-based or framework* or context or mechanism* or activit* or factor* or success* or succeed* or fail* or effective* or fidelity or utility)) 57,550

S3 TI (factor* N1 (hinder* or barrier* or facilitat* or mediat* or moderat* or promot*)) OR AB (factor* N1 (hinder* or barrier* or facilitat* or mediat* or moderat* or promot*)) 3,245

S4 TI (Quality N1 (improv* or management)) OR AB (Quality N1 (improv* or management)) 12,679

S5 TI ("process measure*" or "process assessment*" or "process evaluation*") OR AB ("process measure*" or "process assessment*" or "process evaluation*") 1,275

S6 TI ((complian* or comply or complie* or adhere*) N1 guideline*) OR AB ((complian* or comply or complie* or adhere*) N1 guideline*)92

S7 S1 OR S2 OR S3 OR S4 OR S5 OR S6 85,098

S8 TI ("rapid response system*" or RRS or "rapid response team*" or RRT or "medical emergency team*" or "medical response team*" or "emergency medical team*" or "patient-at-risk team*" or "critical care outreach" or "intensive care outreach") OR AB ("rapid response system*" or RRS or "rapid response team*" or RRT or "medical emergency team*" or "medical response team*" or "emergency medical team*" or "patient-at-risk team*" or "critical care outreach" or "intensive care outreach") 47

S9 TI ("early warning" or "track and trigger" or "warning score*" or "warning system*") OR AB ("early warning" or "track and trigger" or "warning score*" or "warning system*") 483

S10 TI (deteriorat* N2 ("early detection" or "early recognition")) OR AB (deteriorat* N2 ("early detection" or "early recognition")) 1

S11 S8 OR S9 OR S10 531

S12 S7 AND S11 70

S13 S7 AND S11, Limiters - Date Published: 20110101-20231231 46

S14 TI metaanalys* OR meta-analys* or "review" 26,449

S15 S13 AND S14 2

S16 S13 not S15 44

Medication Reconciliation:

Ovid MEDLINE(R) and Epub Ahead of Print, In-Process, In-Data-Review & Other Non-Indexed Citations and Daily <1946 to March 15, 2023> Search Date 16 March 2023

1 implementation science/ 1224

2 Quality Improvement/ 32098

3 Process Assessment, Health Care/ 4981

4 implement*.ti. 72725

5 ((implement* or improv*) adj3 (science* or research* or strateg* or process* or evidence-based or framework* or context or mechanism* or activit* or factor* or success* or succeed* or fail* or effective* or fidelity or utility)).ti,ab,kf. 359686

6 (factor* adj2 (hinder* or barrier* or facilitat* or mediat* or moderat* or promot*)).ti,ab,kf. 55827

7 (Quality adj2 (improv* or management)).ti,ab,kf. 178084

8 (process measure* or process assessment* or process evaluation*).ti,ab,kf. 9010

9 ((complian* or comply or complie* or adhere*) adj2 guideline*).ti,ab,kf. 8237

10 1 or 2 or 3 or 4 or 5 or 6 or 7 or 8 or 9 640054

11 Medication Reconciliation/ 1529

12 ((medication or medicine*) adj2 (reconciliation* or conciliation* or discrepanc*)).ti,ab,kf. 2334

13 (medication list* adj2 (accurac* or inaccurac* or correctness or incorrectness or completeness or incompleteness)).ti,ab,kf. 38

14 best possible medication history.ti,ab,kf. 113

15 11 or 12 or 13 or 14 2912

16 10 and 15 662

17 limit 16 to yr="2011 -Current" 605

18 limit 17 to (meta analysis or "review" or "systematic review") 70

19 17 not 18 535

https://ovidsp.ovid.com/ovidweb.cgi?T=JS&NEWS=N&PAGE=main&SHAREDSEARCHID=4qsPC4Ixg3tOGmRS1utlASaKGxXaLl0g45c2xLAXy9xDd5DKwhONhZovoc7gNr5cK

Embase (Ovid) <1974 to 2023 March 15>; Search Date 16 March 2023

1 implementation science/ 4286

2 total quality management/ 89035

3 implement*.ti. 99653

4 ((implement* or improv*) adj3 (science* or research* or strateg* or process* or evidence-based or framework* or context or mechanism* or activit* or factor* or success* or succeed* or fail* or effective* or fidelity or utility)).ti,ab,kf. 481616

5 (factor* adj2 (hinder* or barrier* or facilitat* or mediat* or moderat* or promot*)).ti,ab,kf. 68937

6 (Quality adj2 (improv* or management)).ti,ab,kf. 272538

7 (process measure* or process assessment* or process evaluation*).ti,ab,kf. 12126

8 ((complian* or comply or complie* or adhere*) adj2 guideline*).ti,ab,kf. 14396

9 1 or 2 or 3 or 4 or 5 or 6 or 7 or 8 891438

10 medication therapy management/ 14642

11 ((medication or medicine*) adj2 (reconciliation* or conciliation* or discrepanc*)).ti,ab,kf. 5249

12 (medication list* adj2 (accurac* or inaccurac* or correctness or incorrectness or completeness or incompleteness)).ti,ab,kf. 61

13 best possible medication history.ti,ab,kf. 222

14 10 or 11 or 12 or 13 16306

15 9 and 14 3157

16 limit 15 to conference abstract 1297

17 15 not 16 1860

18 limit 17 to yr="2011 -Current" 1750

19 limit 18 to "review" 271

20 (meta-analysis or metaanalysis).ti. 217131

21 18 and 20 17

22 19 or 21 275

23 18 not 22 1475

https://ovidsp.ovid.com/ovidweb.cgi?T=JS&NEWS=N&PAGE=main&SHAREDSEARCHID=2zkIfR26KlrRq0GyJlCtiLiVWmuT9OUzBV2AUsShV0ephOmX48zfHS9QLxtK2vUv0

APA PsycInfo (Ovid) <1806 to March Week 1 2023> ; Search Date 16 March 2023

1 implement*.ti. 23586

2 ((implement* or improv*) adj3 (science* or research* or strateg* or process* or evidence-based or framework* or context or mechanism* or activit* or factor* or success* or succeed* or fail* or effective* or fidelity or utility)).tw. 98083

3 (factor* adj2 (hinder* or barrier* or facilitat* or mediat* or moderat* or promot*)).tw. 17207

4 (Quality adj2 (improv* or management)).tw. 30346

5 (process measure* or process assessment* or process evaluation*).tw.3576

6 ((complian* or comply or complie* or adhere*) adj2 guideline*).tw. 948

7 1 or 2 or 3 or 4 or 5 or 6 156074

8 ((medication or medicine*) adj2 (reconciliation* or conciliation* or discrepanc*)).tw. 198

9 (medication list* adj2 (accurac* or inaccurac* or correctness or incorrectness or completeness or incompleteness)).tw. 3

10 best possible medication history.tw. 4

11 8 or 9 or 10 199

12 7 and 11 39

13 limit 12 to ("0200 book" or "0240 authored book" or "0280 edited book" or "0300 encyclopedia" or "0400 dissertation abstract") 7

14 12 not 13 32

15 limit 14 to yr="2011 -Current" 26

16 limit 15 to "reviews (maximizes specificity)" 3

17 15 not 16 23

https://ovidsp.ovid.com/ovidweb.cgi?T=JS&NEWS=N&PAGE=main&SHAREDSEARCHID=2klyGkEWgIzexryVWyk3cQ4uHOulpDpKuQOKEuMWLq5pcDqb24XTg3XjERZjjCplI

CINAHL- EBSCOhost Research Databases, Advanced Search, 1981 - current
Search Date 16 March 2023

S1 (MH "Implementation Science") 827

S2 (MH "Quality Improvement") 66,517

S3 (MH "Process Assessment (Health Care)") 4,909

S4 TI implement* 45,429

S5 TI ((implement* or improv*) N2 (science* or research* or strateg* or process* or evidence-based or framework* or context or mechanism* or activit* or factor* or success* or succeed* or fail* or effective* or fidelity or utility)) OR AB ((implement* or improv*) N2 (science* or research*

or strateg* or process* or evidence-based or framework* or context or mechanism* or activit* or factor* or success* or succeed* or fail* or effective* or fidelity or utility)) 123,993

S6 TI (factor* N1 (hinder* or barrier* or facilitat* or mediat* or moderat* or promot*)) OR AB (factor* N1 (hinder* or barrier* or facilitat* or mediat* or moderat* or promot*)) 11,819

S7 TI (Quality N1 (improv* or management)) OR AB (Quality N1 (improv* or management)) 76,190

S8 TI ("process measure*" or "process assessment*" or "process evaluation*") OR AB ("process measure*" or "process assessment*" or "process evaluation*") 4,853

S9 TI ((complian* or comply or complie* or adhere*) N1 guideline*) OR AB ((complian* or comply or complie* or adhere*) N1 guideline*)3,548

S10 S1 OR S2 OR S3 OR S4 OR S5 OR S6 OR S7 OR S8 OR S9 276,461

S11 (MH "Medication Reconciliation") 2,137

S12 TI ((medication or medicine*) N1 (reconciliation* or conciliation* or discrepanc*)) OR AB ((medication or medicine*) N1 (reconciliation* or conciliation* or discrepanc*)) 1,674

S13 TI ("medication list*" N1 (accurac* or inaccurac* or correctness or incorrectness or completeness or incompleteness)) OR AB ("medication list*" N1 (accurac* or inaccurac* or correctness or incorrectness or completeness or incompleteness)) 27

S14 TI "best possible medication history" OR AB "best possible medication history" 52

S15 S11 OR S12 OR S13 OR S14 2,885

S16 S10 AND S15 659

S17 S10 AND S15,

Limiters - Published Date: 20110101-20231231 552

S18 Limiters - Publication Type: Meta Analysis, Meta Synthesis, Review, Systematic Review 512,729

S19 S17 AND S18 39

S20 S17 NOT S19 513

Web of Science Search Strategy (v0.1) , Database: Web of Science Core Collection (- WOS.SCI: 1945 to 2023, - WOS.AHCI: 1975 to 2023, - WOS.ESCI: 2018 to 2023, - WOS.SSCI: 1956 to 2023), Clarivate; Search Date 16 March 2023

1: TS=((medication or medicine*) NEAR/1 (reconciliation* or conciliation* or discrepanc*)) Results: 2513

2: TS=(("medication list*") NEAR/1 (accurac* or inaccurac* or correctness or incorrectness or completeness or incompleteness)) Results: 34

3: TS=("best possible medication history") Results: 94

4: ((#1) OR #2) OR #3 Results: 2538

5: TI=(implement*) Results: 167943

6: TS=((implement* or improv*) NEAR/2 (science* or research* or strateg* or process* or evidence-based or framework* or context or mechanism* or activit* or factor* or success* or succeed* or fail* or effective* or fidelity or utility)) Results: 637263

7: TS=(factor* NEAR/1 (hinder* or barrier* or facilitat* or mediat* or moderat* or promot*)) Results: 68328

8: TS=(Quality NEAR/1 (improv* or management)) Results: 249926

9: TS=("process measure*" or "process assessment*" or "process evaluation*") Results: 12759

10: TS=((complian* or comply or complie* or adhere*) NEAR/1 guideline*) Results: 8732

11: (((((#5) OR #6) OR #7) OR #8) OR #9) OR #10 Results: 1062466

12: (#4 AND #11) AND ((PY==("2023" OR "2022" OR "2021" OR "2020" OR "2019" OR "2018" OR "2017" OR "2016" OR "2015" OR "2014" OR "2013" OR "2012" OR "2011")) NOT (DT==("MEETING ABSTRACT" OR "PROCEEDINGS PAPER" OR "REVIEW"))) Results: 377

13: (#4 AND #11) AND ((PY==("2023" OR "2022" OR "2021" OR "2020" OR "2019" OR "2018" OR "2017" OR "2016" OR "2015" OR "2014" OR "2013" OR "2012" OR "2011")))
Results: 472

14: (#4 AND #11) AND ((PY==("2023" OR "2022" OR "2021" OR "2020" OR "2019" OR "2018" OR "2017" OR "2016" OR "2015" OR "2014" OR "2013" OR "2012" OR "2011"))) and Review Article (Document Types) Results: 48

Comments: total number of hits = 48 reviews (line 14) + 377 different types of articles (but not reviews, meeting abstracts (line 12).

Database – ERIC (EBSCOhost Research Databases), 1966 to current

Search Date 16 March 2023

S1 TI implement* 19,089

S2 TI ((implement* or improv*) N2 (science* or research* or strateg* or process* or evidence-based or framework* or context or mechanism* or activit* or factor* or success* or succeed* or fail* or effective* or fidelity or utility)) OR AB ((implement* or improv*) N2 (science* or research* or strateg* or process* or evidence-based or framework* or context or mechanism* or activit* or factor* or success* or succeed* or fail* or effective* or fidelity or utility)) 57,550

S3 TI (factor* N1 (hinder* or barrier* or facilitat* or mediat* or moderat* or promot*)) OR AB (factor* N1 (hinder* or barrier* or facilitat* or mediat* or moderat* or promot*)) 3,245

S4 TI (Quality N1 (improv* or management)) OR AB (Quality N1 (improv* or management)) 12,679

S5 TI ("process measure*" or "process assessment*" or "process evaluation*") OR AB ("process measure*" or "process assessment*" or "process evaluation*") 1,275

S6 TI ((complian* or comply or complie* or adhere*) N1 guideline*) OR AB ((complian* or comply or complie* or adhere*) N1 guideline*)92

S7 S1 OR S2 OR S3 OR S4 OR S5 OR S6 85,098

S8 TI ((medication or medicine*) N1 (reconciliation* or conciliation* or discrepanc*)) OR AB ((medication or medicine*) N1 (reconciliation* or conciliation* or discrepanc*)) 2

S9 TI ("medication list*" N1 (accurac* or inaccurac* or correctness or incorrectness or completeness or incompleteness)) OR AB ("medication list*" N1 (accurac* or inaccurac* or correctness or incorrectness or completeness or incompleteness)) 24

S10 TI "best possible medication history" OR AB "best possible medication history" 51

S11 S8 OR S9 OR S10 2

S12 S7 AND S11 1

S13 S7 AND S11, Limiters - Publication Type: Dissertations/Theses (All) 1

Comment: dissertations are excluded, therefore zero hits from this database

The abbreviated terms “Med Rec” or “MedRec” were added to the search strategy on 15th March 2024 and compared with the original searches for Medline and EMBASE. No new relevant papers were found.
