# Supplementary material for: Rapid response systems, antibiotic stewardship and medication reconciliation: a scoping review on implementation factors, activities and outcomes
Source: BMJ Qual Saf. 2024 Jun 6;34(4):e017185. doi: 10.1136/bmjqs-2024-017185 (PMC12013571; doi:10.1136/bmjqs-2024-017185)
Supplement: online supplemental material 2 [file bmjqs-34-4-s002.pdf]

SUPPLEMENTARY MATERIAL 2: ELIGIBILITY CRITERIA

A report will be included in the review if it meets all the following inclusion criteria:

1. Is an original research study or quality improvement report published in a peer reviewed journal.

2. Reports investigations on implementation of antibiotic stewardship, rapid response systems/early warning scores or medication reconciliation in line with the international models/recommendations.

3. Describes the safety practice in enough detail to enable assessment of compliance to the practice as described in guidelines.

4. Is conducted within human health care organizations providing somatic or mental health care.

5. Describes the implementation activities in enough detail for us to be able to assess them. (Any information on concrete, planned activities to facilitate implementation)

6. Describes context factors that helped (facilitators) or hindered (barriers) implementation, either systematically examined and supported with data or hypothesized in the context of an implementation evaluation.

7. Reports implementation outcomes enabling us to identify successful or failed implementation efforts.

8. Is published in 2011 or more recently.

A report will be excluded if it meets one or more of the following criteria:

1. Investigates and reports only effects at patient- or population level without any information about implementation outcome or contextual factors' impact.

2. Does not describe original empirical results of an implementation study or quality improvement effort (e.g. white papers and conference abstracts)

3. Is written in a language other than English.

4a. Specifically for medication reconciliation: Medication reconciliation conducted by pharmacists or non-healthcare services, separate from the ordinary care process (without integration into the clinical workflow).

4b. Specifically for rapid response system: Studies not concerning generic RRS, for instance concerning diagnosis- or procedure-specific rapid response teams such as those assigned for handling sepsis, cardiogenic shock, airway, trauma or extra corporeal membrane oxygenation.

4c. Specifically for antibiotic stewardship program: Studies reporting antimicrobial resistance from laboratory or pharmaceutical perspectives without clinical data.
