# Supplementary material for: Rapid response systems, antibiotic stewardship and medication reconciliation: a scoping review on implementation factors, activities and outcomes
Source: BMJ Qual Saf. 2024 Jun 6;34(4):e017185. doi: 10.1136/bmjqs-2024-017185 (PMC12013571; doi:10.1136/bmjqs-2024-017185)
Supplement: online supplemental material 3 [file bmjqs-34-4-s003.pdf]

SUPPLEMENTARY MATERIAL 3: CALIBRATION SAMPLE

The calibration sample was based on searches in Medline, with search terms limited to subject headings. Thus, we supposed the results contained at least a few possibly relevant records, and as many eligibility criteria as possible could be tested. For each practice the first 30 references, sorted alphabetically by first author, were used.
