# Supplementary figures and images for: Rapid response systems, antibiotic stewardship and medication reconciliation: a scoping review on implementation factors, activities and outcomes
Source: BMJ Qual Saf. 2024 Jun 6;34(4):e017185. doi: 10.1136/bmjqs-2024-017185 (PMC12013571; doi:10.1136/bmjqs-2024-017185)

# SUPPLEMENTARY MATERIAL 4: ILLUSTRATION OF INDUCTIVE CODING PROCESS

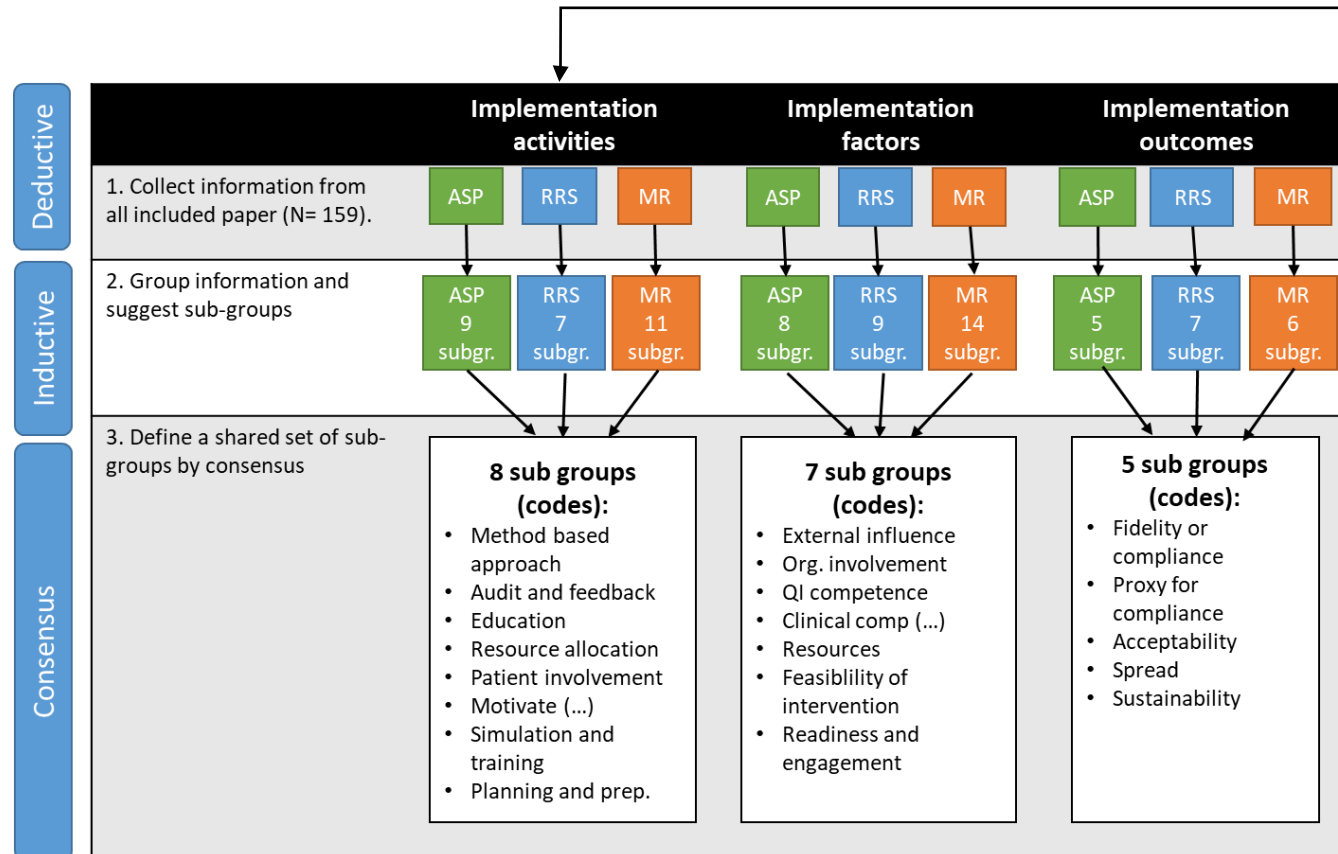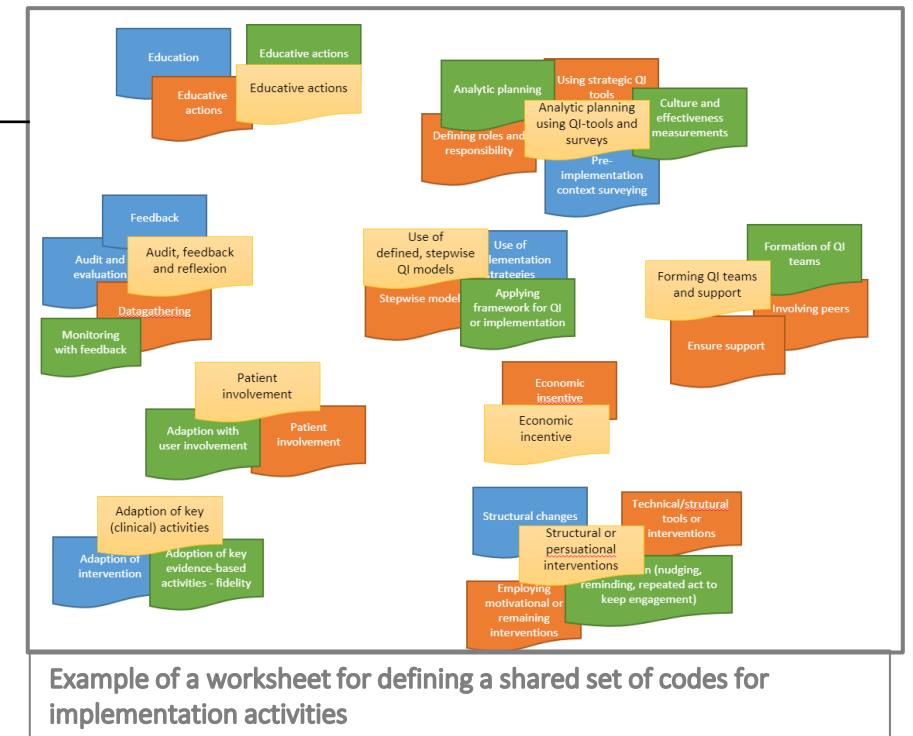

Supplement: online supplemental material 4 [file bmjqs-34-4-s004.pdf]
