# Supplementary material for: Rapid response systems, antibiotic stewardship and medication reconciliation: a scoping review on implementation factors, activities and outcomes
Source: BMJ Qual Saf. 2024 Jun 6;34(4):e017185. doi: 10.1136/bmjqs-2024-017185 (PMC12013571; doi:10.1136/bmjqs-2024-017185)
Supplement: online supplemental material 5 [file bmjqs-34-4-s005.pdf]

SUPPLEMENTARY MATERIAL 5: LIST OF THE PAPERS INCLUDED IN THE REVIEW.

First author (year)[ref]	Country of origin	Setting	Design	Key findings (implementation activities and outcomes)
**Antibiotic stewardship program (ASP)**				
Agarwal (2021) [1]	India	Hospital	Time series	Total AUR reduced, improved compliance with guidelines. Sustained at 1 year.
Albano (2023) [2]	Italy	Hospital	Pre-post	Reduction in AUR and higher adhesion to guidelines, decrease in costs and an increase in quality of care.
Alghamdi (2021) [3]	Saudi Arabia	Hospital	Case Study	Reduced rates of prescription of broad-spectrum antimicrobials using a top-down, stepwise approach.
Alweis (2014) [4]	USA	Hospital – 1 site internal medicine	Pre-post	Increased adherence rate to guidelines after educational and facilitating interventions.
Ambroggio (2013) [5]	USA	Children's hospital	Time series	Improved the appropriate first-line antibiotic prescribing at admission. Sustained for 3 months.
Baier (2021) [6]	USA	Nursing homes (21 units)	Case control	All cases implemented at least one of three ASP tools, resulting in improved early antibiotic discontinuation.
Bhat (2018)[7]	USA	Neonatal intensive care unit	Time series	Decreased overall AUR, did not reach their goal of 90% adherence to guideline algorithm.
Burgess (2019) [8]	USA	Hospital - 178 units	Pre-post	Improved stewardship activities and compliance to recommendations, after a four phases implementation effort.
Carrara (2022) [9]	Italy	Hospital (Internal Medicine)	Time series	Decreased antibiotic consumption and length of stay, enabling prescribers to judicious use of antimicrobials through active participation.
Chiotos (2022) [10]	USA	Hospital (PICU)	Time series	Reduced overall vancomycin use without evidence of harm after education and consensus building.
Chitalia (2022) [11]	USA	Hospital (paediatric CICU)	Time series	Implementation of a protocol limiting empiric antibiotic courses to 24 hours is feasible.
Cunney (2019) [12]	Ireland	Hospital (paediatric)	Time series	Increased guideline compliance and documentation of indication for antibiotics prescription.

Daggett (2022) [13]	USA	Hospital (2 Paediatric ED)	Time series	Reducing empiric antibiotic prescription after «Safety-net antibiotic prescriptions» in conjunction with parent education
Dehn Lunn (2018) [14]	India	Primary care services	Pre-post	A marked and sustainable decrease of inappropriate antibiotic prescriptions was achieved by implementing a multifaceted intervention comprising audit feedback, group training sessions, one-to-one case-based discussion, guideline development and coding updates.
de Vries (2022) [15]	South-Africa	Primary care services	Pre-post	Peer reviewed audit and feedback is effective to improve antibiotic prescribing. Utilising existing resources and involving multidisciplinary engagement, may be incorporated into existing quality improvement processes at facility level, to ensure sustainable change.
Dube (2023) [16]	USA	Paediatric hospital	Time series	Improved the rate of appropriate duration of antibiotics, applying ASP after identifying drivers and applying QI methodology.
Dukhovny (2019) [17]	USA	146 neonatal intensive care units	Time series	Improved compliance to each of the seven domains in Center for Disease Control and Prevention's core elements and decreased median antibiotic use rate after participating in Choosing Antibiotics Wisely.
Forrest (2021) [18]	USA	Primary care services	Time series	Improved proper antibiotics prescribing and use of watchful waiting after using an antibiotic prescribing tool, team meetings and shared decision aid.
Garcia (2022) [19]	USA	Primary care services	Pre-post, mixed methods	Significant decrease in AUR. Insightful information was collected from the focus groups.
Goff (2022) [20]	Australia	Hospital (paediatric)	Time series	Sustained impact on reducing inappropriate antimicrobial use and expenditure and improving compliance with guidelines.
Graus (2022) [21]	Peru	Hospital (neonatal intensive unit)	Time series	Reduced AUR significantly through a comprehensive approach that included team development, education, culture change and regular reporting.
Hamilton (2018) [22]	Sierra Leone	Hospital (outpatient department)	Pre-post	Implementing an empirical antimicrobial guideline was effective. Other measures were required for sustainable

				change. Projects designed to change practice in low-resource countries should include national staff from the outset to improve sustainability.
Hamner (2022) [23]	USA	Hospital (Childrens Urgent Care Clinic)	Time series	Shortened the antibiotic course significantly by addressing primary drivers uncovered through quality improvement methodology.
Hobday (2018) [24]	UK	Hospital (2 respiratory wards)	Time series	Greatly improved review of antimicrobial therapy which was associated with significantly reduced time on intravenous antibiotics.
Jain (2021) [25]	India	Hospital (NICU)	Time series	Significant reduction of AUR through 6 months through multiple interventions using PDSA methodology.
Johnson (2021) [26]	USA	Hospital (outpatient clinic)	Time series	Significant reduction of AUR through 6 months, most success with targeted education of high prescribers.
Jones (2021) [27]	USA	Hospital (ED)	Case-control	Behavioral feedback with peer comparison can be implemented effectively in the ED to reduce inappropriate prescribing.
Joo (2022) [28]	USA	Hospital (paediatric outpatients)	Time series	Implementation of multiple interventions with increasing levels of reliability improved the overall quality of documentation and increased the appropriate antibiotic prescriptions.
Kahn (2022) [29]	USA	Hospital NICU	Time series	Exceeded predetermined goal (28%) of significantly reducing AUR (34-45%) and demonstrated sustainability, using QI methodology and multiple interventions, including multidisciplinary ASP team and education.
Katz MJ (2022) [30]	USA	Long term care facilities	Pre-post	Reduction in antibiotic use and improved outcomes achieved by applying patient-safety principles, multidisciplinary education, and a multitude of interactive tools aimed at incorporating stewardship principles into daily practice
Katz SE (2022) [31]	USA	Hospital (out-patients)	Pre-post	The bundled implementation strategies led to significant increases in guideline-concordant antibiotic prescribing for all diagnoses.

Kaufman (2017) [32]	Canada	Hospital (1 ED)	Time series	A significant reduction of AUR was achieved by applying a front-line ownership driven quality improvement project.
Keller (2022) [33]	USA	Primary care (389 services)	Time series	A safety program addressed attitudes and culture that challenge judicious antibiotic prescribing and incorporated best practices for the management of common infections. Significant reduction of prescription rates.
Kimball (2021) [34]	USA	Hospital (1 site)	Time series	By applying a QI approach focusing on «Engage, Educate, Execute, Evaluate” a significant and sustainable reduction of broad spectrum antibiotics usage was achieved.
Konda (2021) [35]	India	Hospital (1 women and child hospital)	Pre-post	A QI-project applying Plan- Do- Study- Act cycles to test and adapt solutions proportion of unindicated antibiotic usage decreased from 61% to 27%.
Lamb (2021) [36]	UK	Hospital (surgical patients, 1 site)	Time series, mixed methods	Significant reduction of prolonged antibiotics was achived by a QI intervention with interviews providing insight into common themes and barriers surrounding antibiotic prescribing.
Lindberg (2019) [37]	USA	Hospital	Pre-post, mixed methods	Demonstrated effectiveness of a multifaceted intervention in engaging staff and improving antimicrobial prescribing patterns.
Link (2016) [38]	USA	Primary care services	Pre-post	There was a significant decrease posttraining in the number of immediate prescriptions (from 91.7% pretraining to 29.8% posttraining).
Meyers (2020) [39]	USA	Hospital (4 NICU)	Time series	A QI initiative was conducted focusing on addressing gaps in the core elements of ASP and achieved 43% reduction of AUR.
Moehring (2021) [40]	USA	Hospital (17 community hospitals)	Time series	Network hospitals increased ASP activities, demonstrated decline in AUR over a 42-month period. A consultative network model with access to ASP implementation expertise to support long-term program growth.

Morgan (2021) [41]	USA	Primary care services (21 locations)	Pre-post	A behaviourally enhanced quality improvement intervention reduced inappropriate prescribing, 46% reduction of prescriptions.
Mushtaq (2017) [42]	USA	Hospital (1 long term acute hospital)	Pre-post	45-58% reduction of reserve antibiotics, significant reduction of antibiotic costs after implementation of ASP. Sustainability over a 6 year period.
Nampoothiri (2021) [43]	India	Hospital (1 large teaching hospital)	Time series, mixed methods	Significant and sustainable increase of appropriateness and compliance to guidelines through effective leadership, multidisciplinary ASP with clinical pharmacists, audit and feedback.
Nasr (2019) [44]	Quatar	Hospital (1 large tertiary care)	Time series	Slight improvement of prescription practice through effective communication, continuous documentation in records, and repetitive education to promote rational antibiotic prescribing combined with qualitative analysis.
Nedved (2022) [45]	USA	Primary care services (20)	Time series	Overall inappropriate antibiotic prescription rates decreased using PDSA cycles and interventions from an ASP toolkit.
Nkosi (2021) [46]	South-Africa	Hospital (1 ICU)	Case study	The ASP implemented in the facility investigated was sufficient, there are strategies that can be used to optimise the programme and will need regular evaluation.
Pardo (2022) [47]	Belgium	Hospital	Time series	Optimization of surgical antibiotic prophylaxis practices is achievable within a proactive multidisciplinary approach including real-time pharmaceutical interventions in the operating area and in the care unit.
Quintos-Alagheband (2017) [48]	USA	Hospital (paediatric unit)	Time-series	Successful reduction of inappropriate antibiotic use by focusing on standardizing care among different private paediatricians in the community.
Quiros (2022) [49]	Latin America (9 countries)	Hospital (77 ICUs)	Pre-post	Units with high scores on an ASP Core Elements checklist were most successful in implementing the ASP with better outcomes.

Raybardhan (2020) [50]	Canada	Hospital	Time series	A downward trend of AUR was measured after implementing a nurse prompt for appropriate use of antibiotics.
Schmid (2022) [51]	Germany	Hospital (ICU)	Time series	ASP with interprofessional collaboration strategies and education Optimized antimicrobial treatment and improves economic outcome during 5 years.
Sharma (2021) [52]	India	Hospital (maternal care)	Time series	10% reduction in AUR, sustained improvement in the following months, aligning international guidelines and local challenges, sustaining involve continuous feedback to ensure engagement of all stakeholders
Shukla (2020) [53]	USA	Hospital (NICU)	Time series	Substantial reduction of AUR sustained over time following a multifaceted IQ initiative adapting to the local context.
Sikkens (2017) [54]	Netherlands	Hospital (2 hospitals 7 departments)	Pre-post	Use of a behavioural approach preserving prescriber autonomy resulted in an increase in antimicrobial appropriateness sustained for at least 12 months
Singer (2022) [55]	Canada	Primary care services (46 units)	RCT	Significant reduction of antibiotic prescription rates >12 months by use of a behavioural approach preserving prescriber autonomy.
Sloane (2020) [56]	USA	Long term facilities (27 community nursing homes)	Time series	AUR reduced significantly with sustainability through successfully dissemination of ASP through either nursing home administration or medical provider groups.
Tang (2019) [57]	USA	Hospital (3 medical services)	Pre-post	AUR was reduced applying multidisciplinary, frontline provider–driven approaches to ASP
Taylor (2021) [58]	USA	Childrens hospital	Time series	Intermittent education and audit and feedback were associated with reduced misuse of broad-spectrum antibiotics.
Tischendorf (2020) [59]	USA	Hospital (2 units)	Time series, mixed methods	Unwanted antibiotic usage was reduced by applying a framework for successful ASP informed by perceptions of frontline providers
Tonazzi (2022) [60]	USA	Primary care services (1 urgent care clinic)	Pre-post	Success in meeting the goal, reduced AUR, was a result of team and patient engagement strategies.

van Buul (2015) [61]	Netherlands	Long term facilities (10 nursing homes)	Case-control, mixed methods	The approach, or the way it was applied, was not effective in improving antibiotic prescribing behavior. Drawing prescribers' attention to prescribing behavior and monitoring activities, and increasing use of diagnostic resources may be promising interventions.
van den Bergh (2020) [62]	South-Africa	Hospital (39 Sites)	Pre-post	Non-specialised pharmacists in public and private hospitals implemented stewardship interventions and achieved improved compliance to guidelines.
Vaughn (2022) [63]	USA	Hospital (41 sites)	Time series	A significant increase of timely antibiotic usage was achieved through a QI program focusing on benchmarking, sharing best practices, and pay-for-performance incentives.
Verma (2019) [64]	India	Hospital (1 site)	Pre-post	Improvement in antibiotic usage from multiple interventions including audit/feedback and education.
Wathne (2018) [65]	Norway	Hospital (8 wards, 3 hospitals)	RCT	Reduced AUR associated with effect of behaviour change interventions with stakeholder involvement and local target setting
Wolf (2022) [66]	USA	Hospital (1 Children primary care clinic – acute otitis media)	Time series	Increased adherence to guidelines from 78% to 90% after introducing a note template with clinical decision support and provider educational sessions.
Woods-Hill (2022) [67]	USA	Hospital (14 PICU)	Time series	Significant reduction of bloodculture sampling and AUR when implementing a structured ASP including decision support tools.
Yadav (2019) [68]	USA	Hospital 9 sites	RCT	Significant reduction of AUR in both arms (2 ASP approaches, one incorporating nudging). Non-significant effects of specific elements compared.
Yadav (2020) [69]	USA	Hospital 9 sites	Pre-post, mixed methods	An implementation science approach including a preimplementation phase to assess barriers and facilitators to implementing antibiotic stewardship interventions by key stakeholders, with adaptation of the intervention components to the local site and setting followed by an implementation phase led to successful uptake of the intervention.

Yam (2012) [70]	USA	Hospital (1 hospital)	Time series	The implementation of a pharmacy-directed ASP program involving the use of telemedicine technology led to reduced antibiotic costs and decreased indicators of antimicrobial resistant bacteria.
Yeo (2016) [71]	UK	Hospital (1 respiratory unit)	Pre-post	Increased antibiotic prescription compliance to guidelines by applying interventions bringing about a change in personal values, behaviour and individual practice.
Zimmerman (2014) [72]	USA	Long term facilities (12 nursing homes)	Case-control	The QI program reduced the number of prescriptions and the outcomes could be attributed to the commitment of the providers; outreach to providers and staff; and a focus on common clinical situations in which antibiotics are generally not indicated.
**Medication Reconciliation (MedRec)**				
Al Garsan (2021) [73]	USA	Emergency department	Pre-post	More patients received medication reconciliation after an educational intervention for health care teams.
Dos Santos Alcantara (2021) [74]	Brazil	Paediatric ward	Time series	Completeness of MedRec during hospital stay improved after using the "Model for Improvement" methodology.
Almidani (2015) [75]	Saudia Arabia	Paediatric ward (5 units)	Time series	Improved compliance to the MedRec process at admission after introducing educational and monitoring system.
Botros (2019) [76]	UK	Surgical department (2 units initially, spread to other eight units)	Time series	Accuracy of discharge prescription improved using improvement methodologies.
Bruce (2016) [77]	Scotland	General Practice (200 GP practices)	Time series	Improved and sustained compliance to the Scottish Safety in Primary Care Medication Reconciliation care bundle.
Carson (2019) [78]	Canada	2 Hospitals and 5 long-term care homes	Time series	Decreased medication incidents and improved MedRec completion
Curatolo (2015) [79]	France	Surgery department	Time series	Reduced unintended medication discrepancies by implementing, optimizing and sustaining MedRec.

Dabrowski (2021) [80]	New Zealand	Hospital	Time series	Improved rate of completed MedRec after education, standardisation and redesign of process.
El Dannan (2021) [81]	Abu Dhabi	Paediatric department	Time series	Compliance to transfer MedRec unchanged after education and monitoring, improved but not sustained after leadership support, and finally, improved and sustained after adaption of a change model.
Desai (2021) [82]	USA	Hospital with 135 ambulatory practices	Case study	Increased rates of ambulatory MedRec by building an infrastructure focusing on safety reporting, culture and management.
Doolub (2017) [83]	UK	Respiratory ward	Pre-post	Improved rates of MedRec within 24 hours of admission and prior to discharge
Evans (2011) [84]	USA	Hospital (5 units)	Time series	Increased compliance to an on-admission MedRec by a "hard-stop" intervention. Sustained for 6 months.
Harper (2021) [85]	USA	Family medicine residences (4 units)	Time series	Increased percentage of physician-documented MedRec after a team-based care approach. Sustained 1 year after.
Johnson (2018) [86]	USA	Paediatric wards (4 units)	Time series	Increased percentage of patients with MedRec after first, education, and later automated e-mail reminder. Sustained for one year.
Keogh (2016) [87]	USA	Hospital ambulatory practices (148 units)	Time series	Medications reconciled more often during the first twelve months of a financial incentive program.
Kern (2017) [88]	USA	Ambulatory practice for patients with respiratory diseases	Time series	Electronic attestation that MedRec was completed increased after interdisciplinary interventions and changes to the electronic health record (EHR)
Koehl (2021) [89]	USA	Emergency department	Pre-post	Decreased occurance of unintentional discrepancies after expanding the pharmacists' role in the MedRec process.
Kyi (2019) [90]	USA	Hospital	Time series	Increased percentage of completed MedRec using reminders and made changes to the EHR.
Marvin (2016) [91]	UK	Hospital	Time series	Increased percentage of reliable current medication list in patients' discharge documentation after education, introduction of templates and co-design with patient representatives.

Mulligan (2020) [92]	Canada	Oncology clinic	Time series	Improved completion of MedRec after introducing a “closed-loop” system for communicating and understanding failure.
Neufeld (2013) [93]	USA	Outpatient pain clinic	Time series	Increased compliance to MedRec after giving instructions and public recognition of improvement per resident. Sustained for 10 months.
Paton (2011) [94]	UK	Mental health services (42 Trusts)	Multi case study	Modest improvement in percentage of patient where two or more sources of information were consulted about current medicines after an audit-based national QI program.
Phillips (2022) [95]	USA	Academic center and nursing facility	Pre-post	Minimal improvement to an already high MedRec compliance rate.
Presley (2020) [96]	USA	3 Veteran Affairs Hospitals	Case-control	Unintentional medication discrepancies increased for one site and decreased for another after introducing toolkit interventions. No changes to the control site.
Rappaport (2011) [97]	USA	Paediatric hospital (Childrens’ care network)	Retrospective time series	MedRec performance improved, with variation according to practice location, after changes to the EPR, education, provider compliance reports and financial incentives.
Rungvivatjarus (2020) [98]	USA	Paediatric hospital	Time series	MedRec completion rate increased after EPR redesign, clarification of physicians’ role, training, alerts and weekly reports.
Schnipper (2021) [99]	USA	17 Hospitals	Multi case study	Decline in medication discrepancy rate after introducing a refined MARQUIS toolkit
Taha (2016) [100]	United Arab Emir	Medicine department	Time series	Improved admission MedRec compliance after introducing a computerized decision support system. Sustained for four months.
Trivedi (2020) [101]	UK	Paediatric ward	Time series	MedRec completion before mid-day the day after admission improved after educative, awareness and patient/parents involvement interventions.
Vejar (2015) [102]	USA	Geriatric primary care services	Pre-post	Improved MedRec documentation after interventions focusing on educating care providers and patients on medication management.

White (2011) [103]	USA	Paediatric medical center	Time series	Increased rates for MedRec completion within 24 hours of admission after interventions focusing on leadership, support, safety culture, technical feasibility and clarifying roles. Sustained for 27 months.
**Rapid Response Systems (RRS)**				
Acorda (2022) [104]	USA	Paediatric hospital	Time series	Reduction of deficiencies in predefined subset of RRT events. Sustainment at 5 years.
Agulnik (2017) [105]	Guatemala	Paediatric oncology hospital	Pre-post	Full compliance with PEWS documentation and low error rates. Decrease in clinical deterioration events and PICU utilization.
Agulnik (2022)* [106]<br>Agulnik (2022)* [107]<br>Mirochnik (2022)* [108]	Latin America	36 paediatric oncology centers	Time series, mixed methods	Low PEWS error rates after locally adapted implementation using knowledge-to-action framework.
Aitken (2015) [109]	Australia	Tertiary hospital	Pre-post	Reduction of patients meeting call criteria without current treatment. No significant change in CA or ICU transfer rates. Staff satisfaction with the RRS was high.
Allen (2022)*[110]<br>Allen (2022)* [111]	UK	Two general and two tertiary hospitals	Multi case study	Decline in the adverse event rate from implementation of PEWS improvement programme with locally adapted initiatives and structured support.
Almblad (2018) [112]	Sweden	Paediatric hospital	Case study	Implementation using theoretical framework resulted in frequent PEWS use, but incomplete documentation and significant variation in adherence to guidelines. Survey on work context suggest impact on adherence.
Almeida (2018) [113]	Brazil	Tertiary hospital	Time series	Successful RRS implementation using PDSA cycles, reducing ICU waiting time and increasing provision of palliative care.
Al-Qahtani (2013) [114]	Saudi Arabia	Tertiary hospital	Pre-post	Successful implementation using PDSA and simulation training, achieving low response time and decreased non-ICU CA.

Badr (2021) [115]	Egypt	Hospital	Pre-post	Educational measures to implement NEWS and response protocol. Increased frequency of vital signs and medical reviews, decreased CA and ICU transfers.
Bedoya (2019) [116]	USA	Tertiary and community hospital	Pre-post	Automated alerts ignored 86% of the time after implementation of electronic EWS through education and follow-up meetings.
Beitler (2011)[117]	USA	Tertiary hospital	Pre-post	Widely used RRT after implementation. Significant reduction in non-ICU CA, hospital and non-ICU mortality.
Braaten (2015) [118]	USA	Tertiary hospital	Time series	Increased activation rates and high acceptability after RRS improvement project aimed at presumed barriers.
Bunkenborg (2016) [119]	Denmark	Hospital (4 wards)	Pre-post, mixed methods	High short- and long-term adherence and acceptability from RRS implementation including interprofessional simulation and communication training.
Conway-Habes (2017) [120]	USA	Paediatric hospital (1 unit)	Time series	NEWS documentation increased at 15 weeks after activities based on failure modes and effect analysis and using PDSA.
Danesh (2019) [121]	USA	Hospital	Pre-post	Increased RRT activations and reduced ICU transfers from implementation including education and revision of protocols and staffing.
de Groot (2018) [122]	Netherlands	5 hospitals	Time series, mixed methods	Variable improvement in PEWS documentation and appropriate escalation at 1 year after supported and facilitated implementation.
Dean (2020) [123]	USA	Paediatric hospital	Time series	Multiple activities to improve RRS throughout 4-year period resulted in significantly reduced non-ICU CA and stable RRT activation rate.
Douglas (2016) [124]	USA	Paediatric hospital	Pre-post	Implementation of piloted and adapted RRS significantly increased RRT activations and reduced CA rate.
Dryden-Palmer (2022)* [125]<br>Parshuram (2018)* [126]	International	21 paediatric hospitals	Cluster RCT	Customised, theoretically grounded implementation approach resulted in fulfilled adherence requirements after run-in period, increased vital signs measurements and reduced clinical deterioration rate.
Elliot (2019) [127]	Australia	Tertiary hospital	Pre-post	Collaborative, pragmatic QI approach to implementation including governance structures and continuous

				evaluation led to reduction of serious patient incidents with sustained protocol adherence and high satisfaction.
Ennis (2014) [128]	Ireland	Paediatric ward	Case study	Full compliance with monitoring and escalation protocol at 1 year, using training and continuous audit.
Gallo de Moraes (2018) [129]	USA	Hospital	Pre-post	Successful project to increase primary service presence and code status addressed at RRT activations, using a PDSA approach.
Gill (2018) [130]	Australia	Paediatric hospital	Case study	Low levels of parent awareness and involvement in RRT activations. Initial strategies had primarily targeted policy change without taking into account the need for practice and organisational behaviour changes.
Gill (2019) [131]	Australia	Paediatric hospital	Pre-post, mixed methods	Implementation informed by analysis of identified barriers and facilitators using the Theoretical Domains Framework led to increased parent awareness and parent involvement in escalation.
Hanley (2016) [132]	USA	Hospital	Time series	Successful implementation with reduction in CA after collaborative process of piloting and adaptation of the intervention.
Kotsakis (2011)* [133]<br>Lobos (2010)* [134]	Canada	4 Paediatric Hospitals	Pre-post	High RRS activation rate after standardised, phased, multicenter implementation of RRS using social marketing strategies.
Kukreti (2014) [135]	Canada	Paediatric hospital	Case study	Functioning RRS and satisfied users 4 years after phased implementation with focus on empowerment of bedside staff and periodic surveys.
Leach (2012) [136]	USA	Hospital	Case study	Improved and sustained utilization of nurse-led RRT with proactive rounding at 4 years, using education, allocation of resources and focusing on interprofessional collaboration.
Mackintosh (2011) [137]	UK	2 hospitals	Case study	RRS in use but variable adherence to protocol in two different contexts. Implementation, utilisation and impact of the RRS impacted by contextual features.

Martinez (2022) [138]	USA	21 Hospitals	Case-control	Successful spread to 19 hospitals of predictive modelling RRS using strong governance structures, standardized workflows and continuous performance improvement.
McElroy (2019) [139]	Canada	Hospital	Pre-post, mixed methods	High fidelity and satisfaction levels after multifaceted implementation of tailored PEWS.
McGaughey (2017) [140]	UK	2 hospitals (4 wards)	Multiple case study	Improved EWS compliance rate in some wards. Successful implementation dependent on adopting organizational and cultural changes that facilitated staff empowerment, flexible implementation of protocols and ongoing experiential learning.
Menon (2018) [141]	India	Hospital	Time series	Stable RRT activation rate at 2 years and reduced non-ICU CA after trainings and monthly audits.
Miles (2023) [142]	USA	Hospital	Pre-post	Higher rate of patients experiencing rapid response prior to cardiac arrest after EWS implementation with EHR integration and audit.
Miller (2016) [143]	USA	Hospital	Time series	Decreased response times to alerts through piloting, education and use of champions.
Mills (2021) [144]	Palestine	Paediatric ward	Time series	Improvement in PEWS documentation, frequency and accuracy following training, EHR integration and PDSA-based implementation support.
Munroe (2022) [145]	Australia	Emergency department	Case study	A range of implementation strategies were used but overall uptake of the protocol by emergency staff was poor. Several facilitators and barriers were identified.
Pain (2017) [146]	Australia	225 hospitals	Time series, mixed methods	Significant increase in RRT activation rate from comprehensive, statewide implementation effort with governance, training and evaluation elements.
Parshuram (2011) [147]	Canada	Paediatric Ward	Pre-post	Reduced stat calls to physician and clinical deterioration events after interactive trainings of staff.
Paulson (2020) [148]	USA	21 Hospitals	Pre-post	Improvement of activation, timeliness and palliative care documentation after implementing an automated EWS using staged implementation, adaptation after piloting and strong governance structures.

Raff (2022) [149]	USA	Hospital	Time series	Improved team communications and dynamics during RRT activations, meeting pre-set goals.
Rose (2015) [150]	USA	Hospital	Pre-post	Improvement in documentation of EWS scores after educational intervention.
Rosen (2013) [151]	USA	26 hospitals	Time series	Increased RRS call rates and decreased non-ICU CA and mortality from interhospital collaborative with learning sessions and site visits.
Samim (2020) [152]	India	Hospital	Pre-post	Improved use of EWS after training informed by Healthcare Failure Modes and Effect Analysis.
Sebat (2018) [153]	USA	Hospital	Pre-post	Improved performance of RRS with increase in RRT activations and decrease in CA and mortality after restructuring with expanded administrative involvement.
Spiers (2015) [154]	UK	Two hospitals	Pre-post	Improvement in key RRS indicators using PDSA cycles and monthly audit.
Sridhar (2020) [155]	Rwanda	Hospital	Pre-post	Successful PEWS implementation assessed by written tests, clinical skills assessment and chart reviews.
Stolldorf (2020) [156]	USA	Four hospitals	Multi case study	Sustainability of RRTs optimised through effective operationalization of organisational and project design and implementation factors.
Suhr (2020) [157]	USA	Hospital	Pre-post	Decreased reaction time for RRT and increased ICU treatment for patients having multiple RRT activations, after implementing using educational activities.
Tanguay (2017) [158]	Canada	Hospital	Case study	Successful RRS implementation measured by response time and utilization rate using education and audit/feedback.
van der Fluit (2021) [159]	Brazil	Hospital	Pre-post	Improved EWS scoring from training and motivational activities.
Vandegrift (2021) [160]	USA	Four hospitals	Pre-post	Increase of rapid response system activations, more rapid administration of protocolized interventions, and decreases in CA rate and hospital mortality.
Watson (2021) [161]	UK	Hospital	Pre-post	Improved EWS compliance and accuracy through implementing electronic observation system. Staff found

				the system easy to use and helpful. Significant infrastructure and IT support required.
Williams (2011) [162]	USA	Hospital	Pre-post	Reduced RRT activations, CA and adverse events from implementing automated EWS.
Winterbottom (2021) [163]	USA	Hospital	Time series	Staged restructure of RRS including dedicated staffing and interprofessional simulation resulted in improved program outcomes and positive cultural shift.
Wood (2015) [164]	UK	Hospital	Time series	Improved RRS compliance from improvement project including staff engagement, focused education and individualised feedback.

* indicates multiple papers from same study or project

Abbreviations used: AUR: Antibiotic Usage Rate, QI: Quality Improvement, CICU: Cardiac Intensive Care Unit, CA: cardiac arrest, (P)ICU: Paediatric Intensive Care Unit, (P)EWS: (Paediatric) Early Warning Score, NEWS: National Early Warning Score, EHR: electronic health record, PDSA: Plan-Do-Study-Act,

"Setting" includes information on level of health care (ward, department, hospital/center/nursing home/general practices) and patient group if limited to a specific group (e.g., paediatric, surgical or long-term care patients). "Ward" and "department" is within hospitals if not stated otherwise.

Design is described by one of the following: pre-post, time-series (three or more measurements on a timeline), case study (several methods or dataset revealing information about the implementation process and outcome), multi-case study (case study design including several clinics), case-control (comparing clinics implementing the practice with other clinics) or randomised controlled trial (including cluster-RCT). When one study's design can be described in several ways, we report the design expected to be the strongest.

References:

1. Agarwal S, Patodia J, Mittal J, et al. Antibiotic stewardship in a tertiary care NICU of northern India: a quality improvement initiative. BMJ Open Qual. 2021 Jul;10(Suppl 1):e001470. doi: 10.1136/bmjoq-2021-001470. PMID: 34344741; PMCID: PMC8336190.

2. Albano GD, Midiri M, Zerbo S, et al. Implementation of A Year-Long Antimicrobial Stewardship Program in A 227-Bed Community Hospital in Southern Italy. Int J Environ Res Public Health. 2023 Jan 5;20(2):996. doi: 10.3390/ijerph20020996. PMID: 36673754; PMCID: PMC9859386.

3. Alghamdi S, Berrou I, Bajnaid E, et al. Antimicrobial Stewardship Program Implementation in a Saudi Medical City: An Exploratory Case Study. Antibiotics. 2021; 10(3):280. https://doi.org/10.3390/antibiotics10030280

4. Alweis R, Greco M, Wasser T, et al. An initiative to improve adherence to evidence-based guidelines in the treatment of URIs, sinusitis, and pharyngitis. J Community Hosp Intern Med Perspect. 2014 Feb 17;4(1). doi: 10.3402/jchimp.v4.22958. PMID: 24596644; PMCID: PMC3937558.

5. Ambroggio L, Thomson J, Murtagh Kurowski E, et al. White CM. Quality improvement methods increase appropriate antibiotic prescribing for childhood pneumonia. Pediatrics. 2013 May;131(5):e1623-31. doi: 10.1542/peds.2012-2635. Epub 2013 Apr 15. PMID: 23589819; PMCID: PMC3639461.

6. Baier RR, Jump RLP, Zhang T, et al. Feasibility of a Nursing Home Antibiotic Stewardship Intervention. J Am Med Dir Assoc. 2022 Jun;23(6):1025-1030. doi: 10.1016/j.jamda.2021.08.019. Epub 2021 Sep 7. PMID: 34506771.

7. Bhat R, Custodio H, McCurley C, et al. Reducing antibiotic utilization rate in preterm infants: a quality improvement initiative. J Perinatol. 2018 Apr;38(4):421-429. doi: 10.1038/s41372-018-0041-y. Epub 2018 Feb 2. PMID: 29396511.

8. Burgess LH, Miller K, Cooper M, et al. Phased implementation of an antimicrobial stewardship program for a large community hospital system. Am J Infect Control. 2019 Jan;47(1):69-73. doi: 10.1016/j.ajic.2018.06.009. Epub 2018 Aug 3. PMID: 30082089.

9. Carrara E, Sibani M, Barbato L, et al. How to 'SAVE' antibiotics: effectiveness and sustainability of a new model of antibiotic stewardship intervention in the internal medicine area. Int J Antimicrob Agents. 2022 Nov-Dec;60(5-6):106672. doi: 10.1016/j.ijantimicag.2022.106672. Epub 2022 Sep 11. PMID: 36103917.

11. Chitalia RA, Benscoter AL, Chlebowski MM, et al. Implementation of a 24-hour infection diagnosis protocol in the pediatric cardiac intensive care unit (CICU). Infect Control Hosp Epidemiol. 2023 Aug;44(8):1300-1307. doi: 10.1017/ice.2022.265. Epub 2022 Nov 16. PMID: 36382469.

12. Cunney R, Kirrane-Scott M, Rafferty A, et al. 'Start smart': using front-line ownership to improve the quality of empiric antibiotic prescribing in a paediatric hospital. BMJ Open Qual. 2019 Aug 19;8(3):e000445. doi: 10.1136/bmjoq-2018-000445. PMID: 31523725; PMCID: PMC6711488.

13. Daggett A, Wyly DR, Stewart T, et al. Improving Emergency Department Use of Safety-Net Antibiotic Prescriptions for Acute Otitis Media. Pediatr Emerg Care. 2022 Mar 1;38(3):e1151-e1158. doi: 10.1097/PEC.0000000000002525. PMID: 35226640.

14. Dehn Lunn A. Reducing inappropriate antibiotic prescribing in upper respiratory tract infection in a primary care setting in Kolkata, India. BMJ Open Qual. 2018 Nov 20;7(4):e000217. doi: 10.1136/bmjoq-2017-000217. PMID: 30555928; PMCID: PMC6267302.

15. De Vries E, Johnson Y, Willems B, et al. Improving primary care antimicrobial stewardship by implementing a peer audit and feedback intervention in Cape Town community healthcare centres. S Afr Med J. 2022 Oct 5;112(10):812-818. doi: 10.7196/SAMJ.2022.v112i10.16397. PMID: 36472332.

16. Dube AR, Zhao AR, Odozor CU, et al. Improving Prescribing for Otitis Media in a Pediatric Emergency Unit: A Quality Improvement Initiative. Pediatr Qual Saf. 2023 Jan 16;8(1):e625. doi: 10.1097/pq9.0000000000000625. PMID: 36698438; PMCID: PMC9845014.

17. Dukhovny D, Buus-Frank ME, Edwards EM, et al. A Collaborative Multicenter QI Initiative to Improve Antibiotic Stewardship in Newborns. Pediatrics. 2019 Dec;144(6):e20190589. doi: 10.1542/peds.2019-0589. Epub 2019 Nov 1. PMID: 31676682.

18. Forrest CL, Verzone A. Antibiotic stewardship: Improving patient-centered right care in urgent care using a shared decision aid and 5 Ds tool. J Am Assoc Nurse Pract. 2020 Oct 7;33(12):1265-1272. doi: 10.1097/JXX.0000000000000511. PMID: 33038113.

19. Garcia D, Iversen M, Thompson JA and Johnson RA. Reducing Inappropriate Antibiotic Prescribing for Upper Respiratory Infections. The Journal of Nurse Practioners, 2022. 18 (2022): p. 331-4. https://doi.org/10.1016/j.nurpra.2021.12.002

20. Goff Z, Abbotsford J, Yeoh DK, et al. The Impact of a Multifaceted Tertiary Pediatric Hospital's Antimicrobial Stewardship Service. Pediatr Infect Dis J. 2022 Dec 1;41(12):959-966. doi: 10.1097/INF.0000000000003704. Epub 2022 Sep 14. PMID: 36102734.

21. Graus JM, Herbozo C, Hernandez R, et al. Managing antibiotics wisely in a neonatal intensive care unit in a low resource setting. J Perinatol. 2022 Jul;42(7):965-970. doi: 10.1038/s41372-022-01388-4. Epub 2022 Apr 22. PMID: 35459905; PMCID: PMC9023725.

22. Hamilton D and Bugg I. Improving antimicrobial stewardship in the outpatient department of a district general hospital in Sierra Leone. BMJ Open Qual. 2018 Dec 16;7(4):e000495. doi: 10.1136/bmjoq-2018-000495. PMID: 30623113; PMCID: PMC6307603.

23. Hamner M, Nedved A, Austin H, et al. Improving Duration of Antibiotics for Skin and Soft-tissue Infections in Pediatric Urgent Cares. Pediatrics. 2022 Dec 1;150(6):e2022057974. doi: 10.1542/peds.2022-057974. PMID: 36377382.

24. Hobday D, Choudhury A, Asour A, et al. Delivering the 48-hour antimicrobial review on inpatient drug charts. Br J Hosp Med (Lond). 2018 Mar 2;79(3):163-167. doi: 10.12968/hmed.2018.79.3.163. PMID: 29528731.

25. Jain M, Bang A, Meshram P, et al. Institution of an antibiotic stewardship programme for rationalising antibiotic usage: a quality improvement project in the NICU of a public teaching hospital in rural central India. BMJ Open Qual. 2021 Jul;10(Suppl 1):e001456. doi: 10.1136/bmjoq-2021-001456. PMID: 34344740; PMCID: PMC8336129.

26. Johnson MC, Hulgan T, Cooke RG, et al. Operationalising outpatient antimicrobial stewardship to reduce system-wide antibiotics for acute bronchitis. BMJ Open Qual. 2021 Jul;10(3):e001275. doi: 10.1136/bmjoq-2020-001275. PMID: 34210668; PMCID: PMC8252871.

27. Jones GF, Fabre V, Hinson J, et al. Improving antimicrobial prescribing for upper respiratory infections in the emergency department: Implementation of peer comparison with behavioral feedback. Antimicrob Steward Healthc Epidemiol. 2021 Dec 23;1(1):e70. doi: 10.1017/ash.2021.240. PMID: 36168488; PMCID: PMC9495637.

28. Joo KR, Sandberg K, Albertini B, et al. QI Project Promoting NP Compliance with an AOM Bundle in Pediatric Hospital-owned Retail Clinic. Pediatr Qual Saf. 2022 Mar 30;7(2):e537. doi: 10.1097/pq9.0000000000000537. PMID: 35369407; PMCID: PMC8970111.

29. Kahn DJ, Perkins BS, Barrette CE and Godin R. Reducing Antibiotic Use in a Level III and Two Level II Neonatal Intensive Care Units Targeting Prescribing Practices for Both Early and Late-onset Sepsis: A Quality Improvement Project. Pediatr Qual Saf. 2022 Jun 14;7(3):e555. doi: 10.1097/pq9.0000000000000555. PMID: 35720871; PMCID: PMC9197376.

30. Katz MJ, Tamma PD, Cosgrove SE, et al. Implementation of an Antibiotic Stewardship Program in Long-term Care Facilities Across the US. JAMA Netw Open. 2022 Feb 1;5(2):e220181. doi: 10.1001/jamanetworkopen.2022.0181. PMID: 35226084; PMCID: PMC8886516.

31. Katz SE, Spencer P, Cates J, et al. Improvements in appropriate ambulatory antibiotic prescribing using a bundled antibiotic stewardship intervention in general pediatrics practices. Infect Control Hosp Epidemiol. 2022 Dec;43(12):1894-1900. doi: 10.1017/ice.2021.534. Epub 2022 Jan 31. PMID: 35098913.

32. Kaufman AJ, McCready J, Powis J. Impact of a multifaceted antimicrobial stewardship program: A front-line ownership driven quality improvement project in a large urban emergency department. CJEM. 2017 Nov;19(6):441-449. doi: 10.1017/cem.2017.11. Epub 2017 Apr 12. PMID: 28399946.

33. Keller SC, Caballero TM, Tamma PD, et al. Assessment of Changes in Visits and Antibiotic Prescribing During the Agency for Healthcare Research and Quality Safety Program for Improving Antibiotic Use and the COVID-19 Pandemic. JAMA Netw Open. 2022 Jul 1;5(7):e2220512. doi: 10.1001/jamanetworkopen.2022.20512. PMID: 35793084; PMCID: PMC9260475.

34. Kimball JM, Deri CR, Nesbitt WJ, et al. Development of the Three Antimicrobial Stewardship E's (TASE) Framework and Association Between Stewardship Interventions and Intended Results Analysis to Identify Key Facility-Specific Interventions and Strategies for Successful Antimicrobial Stewardship. Clin Infect Dis. 2021 Oct 20;73(8):1397-1403. doi: 10.1093/cid/ciab430. PMID: 33983389.

35. Konda KC, Singh H, Madireddy A and Poodari MMR. Quality improvement initiative approach to decrease the unindicated usage of antibiotics in a neonatal intensive care unit of a tertiary care teaching hospital in Hyderabad, India. BMJ Open Qual. 2021 Jul;10(Suppl 1):e001474. doi: 10.1136/bmjoq-2021-001474. PMID: 34344742; PMCID: PMC8336182.

36. Lamb G, Phillips G, Charani E, et al. Antibiotic prescribing practices in general surgery: a mixed methods quality improvement project. Infect Prev Pract. 2021 Aug 20;3(3):100166. doi: 10.1016/j.infpip.2021.100166. PMID: 34522879; PMCID: PMC8426558.

37. Lindberg CM, Lindberg CC, D'Agata EMC, et al. Advancing Antimicrobial Stewardship in Outpatient Dialysis Centers Using the Positive Deviance Process. Nephrol Nurs J. 2019 Sep-Oct;46(5):511-518. PMID: 31566346.

38. Link TL, Townsend ML, Leung E, et al. Reducing Inappropriate Antibiotic Prescribing for Adults With Acute Bronchitis in an Urgent Care Setting: A Quality Improvement Initiative. Adv Emerg Nurs J. 2016 Oct/Dec;38(4):327-335. doi: 10.1097/TME.0000000000000122. PMID: 27792075.

39. Meyers JM, Tulloch J, Brown K, et al. A Quality Improvement Initiative To Optimize Antibiotic Use in a Level 4 NICU. Pediatrics. 2020 Nov;146(5):e20193956. doi: 10.1542/peds.2019-3956. Epub 2020 Oct 14. PMID: 33055227.

40. Moehring RW, Yarrington ME, Davis AE, et al. Effects of a Collaborative, Community Hospital Network for Antimicrobial Stewardship Program Implementation. Clin Infect Dis. 2021 Nov 2;73(9):1656-1663. doi: 10.1093/cid/ciab356. PMID: 33904897.

41. Morgan BL, Bettencourt H, and May L. Interrupted time-series analysis to evaluate the impact of a behavioral change outpatient antibiotic stewardship intervention. Antimicrob Steward Healthc Epidemiol. 2021 Nov 2;1(1):e37. doi: 10.1017/ash.2021.203. PMID: 36168485; PMCID: PMC9495536.

42. Mushtaq A, Awali RA, Chandramohan S, et al. Implementing an antibiotic stewardship program at a long-term acute care hospital in Detroit, Michigan. Am J Infect Control. 2017 Dec 1;45(12):e157-e160. doi: 10.1016/j.ajic.2017.07.028. Epub 2017 Oct 12. PMID: 29031431.

43. Nampoothiri V, Sudhir AS, Joseph MV, et al. Mapping the Implementation of a Clinical Pharmacist-Driven Antimicrobial Stewardship Programme at a Tertiary Care Centre in South India. Antibiotics (Basel). 2021 Feb 23;10(2):220. doi: 10.3390/antibiotics10020220. PMID: 33672095; PMCID: PMC7926893.

44. Nasr Z, Babiker A, Elbasheer M, et al. Practice implications of an antimicrobial stewardship intervention in a tertiary care teaching hospital, Qatar. East Mediterr Health J. 2019 Apr 25;25(3):172-180. doi: 10.26719/emhj.18.026. PMID: 31054227.

45. Nedved A, Fung M, Bizune D, et al. A Multisite Collaborative to Decrease Inappropriate Antibiotics in Urgent Care Centers. Pediatrics. 2022 Jul 1;150(1):e2021051806. doi: 10.1542/peds.2021-051806. PMID: 35703030.

46. Nkosi BE and Sibanda S. Evaluating an antimicrobial stewardship programme implemented in an intensive care unit of a large academic hospital, using the RE-AIM framework. S Afr Med J. 2021 Aug 2;111(8):777-782. doi: 10.7196/SAMJ.2021.v111i8.15363. PMID: 35227359.

47. Pardo A, Ntabaza V, Rivolta M, et al. Impact of collaborative physician-pharmacist stewardship strategies on prophylactic antibiotic practices: a quasi-experimental study. Antimicrob Resist Infect Control. 2022 Jul 26;11(1):100. doi: 10.1186/s13756-022-01138-3. PMID: 35883189; PMCID: PMC9315847.

48. Quintos-Alagheband ML, Noyola E, Makvana S, et al. Reducing Antibiotic Use in Respiratory Syncytial Virus-A Quality Improvement Approach to Antimicrobial Stewardship. Pediatr Qual Saf. 2017 Dec 1;2(6):e046. doi: 10.1097/pq9.0000000000000046. PMID: 30229182; PMCID: PMC6132889.

49. Quirós RE, Bardossy AC, Angeleri P, et al. Antimicrobial stewardship programs in adult intensive care units in Latin America: Implementation, assessments, and impact on outcomes. Infect Control Hosp Epidemiol. 2022 Feb;43(2):181-190. doi: 10.1017/ice.2021.80. Epub 2021 Apr 8. PMID: 33829982.

50. Raybardhan S, Kan T, Chung B, et al. Nurse Prompting for Prescriber-Led Review of Antimicrobial Use in the Critical Care Unit. Am J Crit Care. 2020 Jan 1;29(1):71-76. doi: 10.4037/ajcc2020272. PMID: 31968088.

51. Schmid S, Schlosser S, Gülow K, et al. Interprofessional Collaboration between ICU Physicians, Staff Nurses, and Hospital Pharmacists Optimizes Antimicrobial Treatment and Improves Quality of Care and Economic Outcome. Antibiotics (Basel). 2022 Mar 13;11(3):381. doi: 10.3390/antibiotics11030381. PMID: 35326844; PMCID: PMC8944851.

52. Sharma S, Kumari N, Sengupta R, et al. Rationalising antibiotic use after low-risk vaginal deliveries in a hospital setting in India. BMJ Open Qual. 2021 Jul;10(Suppl 1):e001413. doi: 10.1136/bmjoq-2021-001413. PMID: 34344734; PMCID: PMC8336128.

53. Shukla S, Cortez J, Renfro B, et al. Charge Nurses Taking Charge, Challenging the Culture of Culture-Negative Sepsis, and Preventing Central-Line Infections to Reduce NICU Antibiotic Usage. Am J Perinatol. 2022 Jun;39(8):861-868. doi: 10.1055/s-0040-1719079. Epub 2020 Nov 3. PMID: 33142341.

54. Sikkens JJ, van Agtmael MA, Peters EJG, et al. Behavioral Approach to Appropriate Antimicrobial Prescribing in Hospitals: The Dutch Unique Method for Antimicrobial Stewardship (DUMAS) Participatory Intervention Study. JAMA Intern Med. 2017 Aug 1;177(8):1130-1138. doi: 10.1001/jamainternmed.2017.0946. PMID: 28459929; PMCID: PMC5818788.

55. Singer A, Kosowan L, Abrams EM, et al. Implementing an audit and feedback cycle to improve adherence to the Choosing Wisely Canada recommendations: clustered randomized trail. BMC Prim Care. 2022 Nov 26;23(1):302. doi: 10.1186/s12875-022-01912-7. PMID: 36435746; PMCID: PMC9701433.

56. Sloane PD, Zimmerman S, Ward K, et al. A 2-Year Pragmatic Trial of Antibiotic Stewardship in 27 Community Nursing Homes. J Am Geriatr Soc. 2020 Jan;68(1):46-54. doi: 10.1111/jgs.16059. Epub 2019 Jul 18. PMID: 31317534.

57. Tang SJ, Gupta R, Lee JI, et al. Impact of Hospitalist-Led Interdisciplinary Antimicrobial Stewardship Interventions at an Academic Medical Center. Jt Comm J Qual Patient Saf. 2019 Mar;45(3):207-216. doi: 10.1016/j.jcjq.2018.09.002. Epub 2018 Oct 25. PMID: 30482662.

58. Taylor M, Liechti S and Palazzi D. Intermittent Education and Audit and Feedback Reduce Inappropriate Prescribing of Oral Third-Generation Cephalosporins for Pediatric Upper Respiratory Tract Infections. Jt Comm J Qual Patient Saf. 2021 Apr;47(4):250-257. doi: 10.1016/j.jcjq.2020.12.003. Epub 2020 Dec 14. PMID: 33446441.

59. Tischendorf J, Brunner M, Knobloch MJ, et al. Evaluation of a successful fluoroquinolone restriction intervention among high-risk patients: A mixed-methods study. PLoS One. 2020 Aug 25;15(8):e0237987. doi: 10.1371/journal.pone.0237987. PMID: 32841259; PMCID: PMC7446965.

60. Tonazzi S, Prenovost L and Scheuermann S. Delayed antibiotic prescribing to reduce antibiotic use: an urgent care practice change. BMJ Open Qual. 2022 Mar;11(1):e001513. doi: 10.1136/bmjoq-2021-001513. PMID: 35264330; PMCID: PMC8915352.

61. van Buul LW, van der Steen JT, Achterberg WP, et al. Effect of tailored antibiotic stewardship programmes on the appropriateness of antibiotic prescribing in nursing homes. J Antimicrob Chemother. 2015 Jul;70(7):2153-62. doi: 10.1093/jac/dkv051. Epub 2015 Mar 4. PMID: 25745104.

62. van den Bergh D, Messina AP, Goff DA, et al. South African Antibiotic Stewardship Programme Pharmacist Community-acquired Pneumonia Study Alliance. A pharmacist-led prospective antibiotic stewardship intervention improves compliance to community-acquired pneumonia guidelines in 39 public and private hospitals across South Africa. Int J Antimicrob Agents. 2020 Dec;56(6):106189. doi: 10.1016/j.ijantimicag.2020.106189. Epub 2020 Oct 9. PMID: 33045348.

63. Vaughn VM, Gandhi TN, Hofer TP, et al. A Statewide Collaborative Quality Initiative to Improve Antibiotic Duration and Outcomes in Patients Hospitalized With Uncomplicated Community-Acquired Pneumonia. Clin Infect Dis. 2022 Aug 31;75(3):460-467. doi: 10.1093/cid/ciab950. PMID: 34791085; PMCID: PMC9427146.

64. Verma M, Shafiq N, Tripathy JP, et al. Antimicrobial stewardship programme in a trauma centre of a tertiary care hospital in North India: Effects and implementation challenges. J Glob Antimicrob Resist. 2019 Jun;17:283-290. doi: 10.1016/j.jgar.2019.02.020. Epub 2019 Mar 2. PMID: 30836142.

65. Wathne JS, Kleppe LKS, Harthug S, et al. The effect of antibiotic stewardship interventions with stakeholder involvement in hospital settings: a multicentre, cluster randomized controlled intervention study. Antimicrob Resist Infect Control. 2018 Sep 10;7:109. doi: 10.1186/s13756-018-0400-7. PMID: 30214718; PMCID: PMC6131848.

66. Wolf RM, Langford KT and Patterson BL. Improving Adherence to AAP Acute Otitis Media Guidelines in an Academic Pediatrics Practice through a Quality Improvement Project. Pediatr Qual Saf. 2022 Jun 14;7(3):e553. doi: 10.1097/pq9.0000000000000553. PMID: 35720875; PMCID: PMC9197370.

67. Woods-Hill CZ, Colantuoni EA, Koontz DW, et al. Association of Diagnostic Stewardship for Blood Cultures in Critically Ill Children With Culture Rates, Antibiotic Use, and Patient Outcomes: Results of the Bright STAR Collaborative. JAMA Pediatr. 2022 Jul 1;176(7):690-698. doi: 10.1001/jamapediatrics.2022.1024. PMID: 35499841; PMCID: PMC9062771.

68. Yadav K, Meeker D, Mistry RD, et al. A Multifaceted Intervention Improves Prescribing for Acute Respiratory Infection for Adults and Children in Emergency Department and Urgent Care Settings. Acad Emerg Med. 2019 Jul;26(7):719-731. doi: 10.1111/acem.13690. Epub 2019 Jun 19. PMID: 31215721; PMCID: PMC8146207.

69. Yadav K, Stahmer A, Mistry RD and May L. An Implementation Science Approach to Antibiotic Stewardship in Emergency Departments and Urgent Care Centers. Acad Emerg Med. 2020 Jan;27(1):31-42. doi: 10.1111/acem.13873. Epub 2019 Nov 22. PMID: 31625653.

70. Yam P, Fales D, Jemison J, et al. Implementation of an antimicrobial stewardship program in a rural hospital. Am J Health Syst Pharm. 2012 Jul 1;69(13):1142-8. doi: 10.2146/ajhp110512. PMID: 22722593.

71. Yeo JM. Antimicrobial stewardship: Improving antibiotic prescribing practice in a respiratory ward. BMJ Qual Improv Rep. 2016 Jan 14;5(1):u206491.w3570. doi: 10.1136/bmjquality.u206491.w3570. PMID: 26893898; PMCID: PMC4752714.

72. Zimmerman S, Sloane PD, Bertrand R, et al. Successfully reducing antibiotic prescribing in nursing homes. J Am Geriatr Soc. 2014 May;62(5):907-12. doi: 10.1111/jgs.12784. Epub 2014 Apr 2. PMID: 24697789.

73. Al Garsan M, Leon E, Alyami HS, et al. Effectiveness of an educational intervention in improving the patient medication reconciliation in the emergency department. Int J Clin Pract. 2021 Nov;75(11):e14782. doi: 10.1111/ijcp.14782. Epub 2021 Sep 8. PMID: 34482599.

74. Alcântara TDS, Lima HF, Valença-Feitosa F, et al. Development and implementation of a medication reconciliation during pediatric transitions of care in a public hospital. J Am Pharm Assoc (2003). 2022 Jul-Aug;62(4):1400-1406.e3. doi: 10.1016/j.japh.2021.12.009. Epub 2021 Dec 20. PMID: 34998691.

75. Almidani E, Khadawardi E, Alshareef T, et al. Improving Medication Reconciliation compliance at admission: A single department's experience. Int J Pediatr Adolesc Med. 2015 Sep-Dec;2(3-4):141-146. doi: 10.1016/j.ijpam.2015.09.005. Epub 2015 Oct 30. PMID: 30805453; PMCID: PMC6372393.

76. Botros S and Dunn J. Implementation and spread of a simple and effective way to improve the accuracy of medicines reconciliation on discharge: a hospital-based quality improvement project and success story. BMJ Open Qual. 2019 Aug 1;8(3):e000363. doi: 10.1136/bmjoq-2018-000363. PMID: 31428702; PMCID: PMC6683109.

77. Bruce R. Large scale implementation of a medicines reconciliation care bundle in NHS GGC GP practices. BMJ Qual Improv Rep. 2016 Nov 7;5(1):u212988.w6116. doi: 10.1136/bmjquality.u212988.w6116. PMID: 27933147; PMCID: PMC5128764.

78. Carson J, Gottheil S, Lawson S and Rice T. London Transfer Project: Reducing Medication Incidents After Discharge From Hospital to Long-term Care. J Am Med Dir Assoc. 2019 Apr;20(4):481-486. doi: 10.1016/j.jamda.2018.09.037. Epub 2018 Dec 5. PMID: 30528140.

79. Curatolo N, Gutermann L, Devaquet N, et al. Reducing medication errors at admission: 3 cycles to implement, improve and sustain medication reconciliation. Int J Clin Pharm. 2015 Feb;37(1):113-20. doi: 10.1007/s11096-014-0047-2. Epub 2014 Dec 3. PMID: 25468221.

80. Dabrowski PM and Lawrie K. Twelve-week project to improve medication reconciliation at hospitals in Wellington, New Zealand. BMJ Open Qual. 2021 Jun;10(2):e000787. doi: 10.1136/bmjoq-2019-000787. PMID: 34127451; PMCID: PMC8204172.

81. Dannan HE and Ellahham S. Improving Transfer Medication Reconciliation in an Emirati Tertiary Hospital Utilizing the Irish Health Service Executive Model. Am J Med Qual. 2021 Jan-Feb 01;36(1):49-56. doi: 10.1177/1062860620920712. PMID: 32418444.

82. Dannan HE and Ellahham S. Improving Transfer Medication Reconciliation in an Emirati Tertiary Hospital Utilizing the Irish Health Service Executive Model. Am J Med Qual. 2021 Jan-Feb 01;36(1):49-56. doi: 10.1177/1062860620920712. PMID: 32418444.

83. Doolub R. Improving medicines reconciliation rates at Ashford and St. Peter's Hospitals NHS Foundation Trust. BMJ Qual Improv Rep. 2017 Jun 8;6(1):e000064. doi: 10.1136/bmjquality-2017-000064. PMID: 28824809; PMCID: PMC5492467.

84. Evans AS, Lazar EJ, Tiase VL, et al. The role of housestaff in implementing medication reconciliation on admission at an academic medical center. Am J Med Qual. 2011 Jan-Feb;26(1):39-42. doi: 10.1177/1062860610370712. Epub 2010 May 25. PMID: 20501865.

85. Harper PG, Schafer KM, Van Riper K, et al. Team-based approach to improving medication reconciliation rates in family medicine residency clinics. J Am Pharm Assoc (2003). 2021 Jan-Feb;61(1):e46-e52. doi: 10.1016/j.japh.2020.08.007. Epub 2020 Sep 10. PMID: 32919924.

86. Johnson K, Burkett GS, Nelson D, et al. Automated E-mail Reminders Linked to Electronic Health Records to Improve Medication Reconciliation on Admission. Pediatr Qual Saf. 2018 Sep 19;3(5):e109. doi: 10.1097/pq9.0000000000000109. PMID: 30584636; PMCID: PMC6221599.

87. Keogh C, Kachalia A, Fiumara K, et al. Ambulatory Medication Reconciliation: Using a Collaborative Approach to Process Improvement at an Academic Medical Center. Jt Comm J Qual Patient Saf. 2016 Apr;42(4):186-94. doi: 10.1016/s1553-7250(16)42023-4. PMID: 27025579.

88. Kern E, Dingae MB, Langmack EL, et al. Measuring to Improve Medication Reconciliation in a Large Subspecialty Outpatient Practice. Jt Comm J Qual Patient Saf. 2017 May;43(5):212-223. doi: 10.1016/j.jcjq.2017.02.005. Epub 2017 Mar 30. PMID: 28434454.

89. Koehl J, Steffenhagen A and Halfpap J. Implementation and Impact of Pharmacist-Initiated Home Medication Ordering in an Emergency Department Observation Unit. Journal of Pharmacy Practice. 2021;34(3):459-464. doi:10.1177/0897190019879254

90. Kyi HH, Sundus S, Marcus H, et al. Directed intervention to improve the rate of admission medication reconciliation in an acute care hospital. BMJ Open Qual. 2019 Dec 30;8(4):e000784. doi: 10.1136/bmjoq-2019-000784. PMCID: PMC7011894.

91. Marvin V, Kuo S, Poots AJ et al. Applying quality improvement methods to address gaps in medicines reconciliation at transfers of care from an acute UK hospital. BMJ Open. 2016 Jun 9;6(6):e010230. doi: 10.1136/bmjopen-2015-010230. PMID: 27288369; PMCID: PMC4908889.

92. Mulligan E, Tuff LR, Leclair J, et al. Implementation of a closed-loop medication reconciliation process for ambulatory oncology patients at Winchester District Memorial Hospital. Healthc Manage Forum. 2020 Mar;33(2):85-89. doi: 10.1177/0840470419889650. Epub 2019 Dec 20. PMID: 31858820.

93. Neufeld NJ, González Fernández M, Christo PJ and Williams KA. Positive recognition program increases compliance with medication reconciliation by resident physicians in an outpatient clinic. Am J Med Qual. 2013 Jan-Feb;28(1):40-5. doi: 10.1177/1062860612443550. Epub 2012 May 16. PMID: 22605782.

94. Paton C, McIntyre S, Bhatti SF,et al. Medicines Reconciliation on Admission to Inpatient Psychiatric Care: Findings from a UK Quality Improvement Programme. Ther Adv Psychopharmacol. 2011 Aug;1(4):101-10. doi: 10.1177/2045125311417299. PMID: 23983934; PMCID: PMC3736923.

95. Phillips M, Dillaman M, Matuga R, et al. 30-Day Readmission Reduction in a Skilled Facility Population Through Pharmacist-Driven Medication Reconciliation. J Healthc Qual. 2022 May-Jun 01;44(3):152-160. doi: 10.1097/JHQ.0000000000000313. PMID: 35506711.

96. Presley CA, Wooldridge KT, Byerly SH, et al. The Rural VA Multi-Center Medication Reconciliation Quality Improvement Study (R-VA-MARQUIS). Am J Health Syst Pharm. 2020 Jan 8;77(2):128-137. doi: 10.1093/ajhp/zxz275. PMID: 31912884.

97. Rappaport DI, Collins B, Koster A, et al. Implementing medication reconciliation in outpatient pediatrics. Pediatrics. 2011 Dec;128(6):e1600-7. doi: 10.1542/peds.2011-0993. Epub 2011 Nov 28. PMID: 22123872.

98. Rungvivatjarus T, Kuelbs CL, Miller L, et al. Medication Reconciliation Improvement Utilizing Process Redesign and Clinical Decision Support. Jt Comm J Qual Patient Saf. 2020 Jan;46(1):27-36. doi: 10.1016/j.jcjq.2019.09.001. Epub 2019 Oct 22. PMID: 31653526.

99. Schnipper JL, Reyes Nieva H, Mallouk M, et al. Effects of a refined evidence-based toolkit and mentored implementation on medication reconciliation at 18 hospitals: results of the MARQUIS2 study. BMJ Qual Saf. 2022 Apr;31(4):278-286. doi: 10.1136/bmjqs-2020-012709. Epub 2021 Apr 29. PMID: 33927025.

100. Taha H, Abdulhay D, Luqman N and Ellahham S. Improving admission medication reconciliation compliance using the electronic tool in admitted medical patients. BMJ Qual Improv Rep. 2016 Jun 6;5(1):u209593.w4322. doi: 10.1136/bmjquality.u209593.w4322. PMID: 27822371; PMCID: PMC5067707.

101. Trivedi A, Sharma S, Ajitsaria R and Davey NJ. Please reconcile, not wait a while. Arch Dis Child Educ Pract Ed. 2020 Apr;105(2):122-126. doi: 10.1136/archdischild-2018-316356. Epub 2019 May 9. PMID: 31073034.

102. Vejar MV, Makic MB and Kotthoff-Burrell E. Medication management for elderly patients in an academic primary care setting: a quality improvement project. J Am Assoc Nurse Pract. 2015 Feb;27(2):72-8. doi: 10.1002/2327-6924.12121. Epub 2014 May 16. PMID: 24838763.

103. White CM, Schoettker PJ, Conway PH, et al. Utilising improvement science methods to optimise medication reconciliation. BMJ Qual Saf. 2011 Apr;20(4):372-80. doi: 10.1136/bmjqs.2010.047845. Epub 2011 Feb 11. PMID: 21317180.

104. Acorda DE, Bracken J, Abela K, et al. Longitudinal Evaluation of a Pediatric Rapid Response System with Realist Evaluation Framework. Jt Comm J Qual Patient Saf. 2022 Apr;48(4):196-204. doi: 10.1016/j.jcjq.2022.01.004. Epub 2022 Jan 13. PMID: 35181251.

105. Agulnik A, Mora Robles LN, Forbes PW, et al. Improved outcomes after successful implementation of a pediatric early warning system (PEWS) in a resource-limited pediatric oncology hospital. Cancer. 2017 Aug 1;123(15):2965-2974. doi: 10.1002/cncr.30664. Epub 2017 Apr 25. PMID: 28440868.

106. Agulnik A, Ferrara G, Puerto-Torres M, et al. Assessment of Barriers and Enablers to Implementation of a Pediatric Early Warning System in Resource-Limited Settings. JAMA Netw Open. 2022 Mar 1;5(3):e221547. doi: 10.1001/jamanetworkopen.2022.1547. PMID: 35262714; PMCID: PMC8908074.

107. Agulnik A, Gonzalez Ruiz A, Muniz-Talavera H, et al. Model for regional collaboration: Successful strategy to implement a pediatric early warning system in 36 pediatric oncology centers in Latin America. Cancer. 2022 Nov 15;128(22):4004-4016. doi: 10.1002/cncr.34427. Epub 2022 Sep 26. PMID: 36161436; PMCID: PMC9828186.

108. Mirochnick E, Graetz DE, Ferrara G, et al. Multilevel impacts of a pediatric early warning system in resource-limited pediatric oncology hospitals. Front Oncol. 2022 Oct 12;12:1018224. doi: 10.3389/fonc.2022.1018224. PMID: 36313665; PMCID: PMC9597682.

109. Aitken LM, Chaboyer W, Vaux A, et al. Effect of a 2-tier rapid response system on patient outcome and staff satisfaction. Aust Crit Care. 2015 Aug;28(3):107-14; quiz 115. doi: 10.1016/j.aucc.2014.10.044. Epub 2014 Dec 12. PMID: 25498252.

110. Allen D, Lloyd A, Edwards D et al. Development, implementation and evaluation of an evidence-based paediatric early warning system improvement programme: the PUMA mixed methods study. BMC Health Serv Res 22, 9 (2022). https://doi.org/10.1186/s12913-021-07314-2

111. Allen D, Lloyd A, Edwards D et al. Development, implementation and evaluation of an early warning system improvement programme for children in hospital: the PUMA mixed-methods study. Southampton (UK): NIHR Journals Library; 2022 Jan. PMID: 35129935.

112. Almblad AC, Siltberg P, Engvall G and Målqvist M. Implementation of Pediatric Early Warning Score; Adherence to Guidelines and Influence of Context. J Pediatr Nurs. 2018 Jan-Feb;38:33-39. doi: 10.1016/j.pedn.2017.09.002. Epub 2017 Oct 23. PMID: 29167078.

113. Almeida MC, Portela MC, Paiva EP, et al. Implementation of a rapid response team in a large nonprofit Brazilian hospital: improving the quality of emergency care through Plan-Do-Study-Act. Rev Bras Ter Intensiva. 2019 Jun 10;31(2):217-226. doi: 10.5935/0103-507X.20190036. PMID: 31215601; PMCID: PMC6649208. 114. Al-Qahtani, S., et al., Impact of an intensivist-led multidisciplinary extended rapid response team on hospital-wide cardiopulmonary arrests and mortality. Crit Care Med, 2013. 41(2): p. 506-17.

115. Badr MN, Khalil NS and Mukhtar AM. Effect of National Early Warning Scoring System Implementation on Cardiopulmonary Arrest, Unplanned ICU Admission, Emergency Surgery, and Acute Kidney Injury in an Emergency Hospital, Egypt. J Multidiscip Healthc. 2021 Jun 15;14:1431-1442. doi: 10.2147/JMDH.S312395. PMID: 34163171; PMCID: PMC8214550.

116. Bedoya AD, Clement ME, Phelan M, et al. Minimal Impact of Implemented Early Warning Score and Best Practice Alert for Patient Deterioration. Crit Care Med. 2019 Jan;47(1):49-55. doi: 10.1097/CCM.0000000000003439. PMID: 30247239; PMCID: PMC6298839.

117. Beitler JR, Link N, Bails DB, et al. Reduction in hospital-wide mortality after implementation of a rapid response team: a long-term cohort study. Crit Care. 2011;15(6):R269. doi: 10.1186/cc10547. Epub 2011 Nov 15. PMID: 22085785; PMCID: PMC3388666.

118. Braaten JS, deGunst G and Bilys K. Rapidly Increasing Rapid Response Team Activation Rates. Jt Comm J Qual Patient Saf. 2015 Sep;41(9):421-7. doi: 10.1016/s1553-7250(15)41054-2. PMID: 26289237.

119. Bunkenborg G, Poulsen I, Samuelson K, et al. Mandatory early warning scoring--implementation evaluated with a mixed-methods approach. Appl Nurs Res. 2016 Feb;29:168-76. doi: 10.1016/j.apnr.2015.06.012. Epub 2015 Jun 29. PMID: 26856510.

120. Conway-Habes EE, Herbst BF Jr, Herbst LA, et al. Using Quality Improvement to Introduce and Standardize the National Early Warning Score (NEWS) for Adult Inpatients at a Children's Hospital. Hosp Pediatr. 2017 Mar;7(3):156-163. doi: 10.1542/hpeds.2016-0117. PMID: 28232377.

121. Danesh V, Neff D, Jones TL, et al. Can proactive rapid response team rounding improve surveillance and reduce unplanned escalations in care? A controlled before and after study. Int J Nurs Stud. 2019 Mar;91:128-133. doi: 10.1016/j.ijnurstu.2019.01.004. Epub 2019 Jan 12. PMID: 30690288.

122. de Groot JF, Damen N, de Loos E, et al. Implementing paediatric early warning scores systems in the Netherlands: future implications. BMC Pediatr. 2018 Apr 6;18(1):128. doi: 10.1186/s12887-018-1099-6. PMID: 29625600; PMCID: PMC5889599.

123. Dean NP, Ghebremariam E, Szeles R, et al. Late Rescue Collaborative: Reducing Non-ICU Arrests. Pediatr Crit Care Med. 2020 Jun;21(6):513-519. doi: 10.1097/PCC.0000000000002224. PMID: 31851129.

124. Douglas K, Collado JC and Keller S. Implementation of a Pediatric Early Warning Scoring System at an Academic Medical Center. Crit Care Nurs Q. 2016 Oct-Dec;39(4):363-70. doi: 10.1097/CNQ.0000000000000130. PMID: 27575799.

125. Dryden-Palmer K, Berta WB and Parshuram CS. Implementing a complex hospital innovation: conceptual underpinnings, program design and implementation of a complex innovation in an international multi-site hospital trial. BMC Health Serv Res. 2022 Nov 12;22(1):1342. doi: 10.1186/s12913-022-08768-8. PMID: 36371214; PMCID: PMC9652896.

126. Parshuram CS, Dryden-Palmer K, Farrell C, et al. Effect of a Pediatric Early Warning System on All-Cause Mortality in Hospitalized Pediatric Patients: The EPOCH Randomized Clinical Trial. JAMA. 2018 Mar 13;319(10):1002-1012. doi: 10.1001/jama.2018.0948. PMID: 29486493; PMCID: PMC5885881.

127. Elliott R, Martyn L, Woodbridge S, et al. Development and Pragmatic Evaluation of a Rapid Response Team. Crit Care Nurs Q. 2019 Jul/Sep;42(3):227-234. doi: 10.1097/CNQ.0000000000000263. PMID: 31135473.

128. Ennis L. Paediatric early warning scores on a children's ward: a quality improvement initiative. Nurs Child Young People. 2014 Sep;26(7):25-31. doi: 10.7748/ncyp.26.7.25.e478. PMID: 25200240.

129. Gallo de Moraes A, O'Horo JC, Sevilla-Berrios RA, et al. Expanding the Presence of Primary Services at Rapid Response Team Activations: A Quality Improvement Project. Qual Manag Health Care. 2018 Jan/Mar;27(1):50-55. doi: 10.1097/QMH.0000000000000159. PMID: 29280908.

130. Gill FJ, Leslie GD and Marshall AP. Barriers and facilitators to implementing a process to enable parent escalation of care for the deteriorating child in hospital. Health Expect. 2018 Dec;21(6):1095-1103. doi: 10.1111/hex.12806. Epub 2018 Jul 2. PMID: 29962031; PMCID: PMC6250884.

131. Gill FJ, Leslie GD and Marshall AP. Parent escalation of care for the deteriorating child in hospital: A health-care improvement study. Health Expect. 2019 Oct;22(5):1078-1088. doi: 10.1111/hex.12938. Epub 2019 Jul 16. PMID: 31309665; PMCID: PMC6803393.

132. Hanley D, Abele D, Alley AJ, et al. Creating a Culture of Safety Through Integration of an Early Warning System. J Nurs Adm. 2016 Feb;46(2):63-8. doi: 10.1097/NNA.0000000000000296. PMID: 26796820.

133. Kotsakis A, Lobos AT, Parshuram C, et al. Implementation of a multicenter rapid response system in pediatric academic hospitals is effective. Pediatrics. 2011 Jul;128(1):72-8. doi: 10.1542/peds.2010-0756. Epub 2011 Jun 20. PMID: 21690113.

134. Lobos AT, Costello J, Gilleland J, et al. An implementation strategy for a multicenter pediatric rapid response system in Ontario. Jt Comm J Qual Patient Saf. 2010 Jun;36(6):271-80, 241. doi: 10.1016/s1553-7250(10)36043-0. PMID: 20564889.

135. Kukreti V, Gaiteiro R and Mohseni-Bod H. Implementation of a pediatric rapid response team: experience of the Hospital for Sick Children in Toronto. Indian Pediatr. 2014 Jan;51(1):11-5. doi: 10.1007/s13312-014-0323-1. PMID: 24561462.

136. Leach LS, Kagawa F, Mayo A and Pugh C. Improving patient safety to reduce preventable deaths: the case of a California safety net hospital. J Healthc Qual. 2012 Mar-Apr;34(2):64-76. doi: 10.1111/j.1945-1474.2011.00185.x. PMID: 23552203.

137. Mackintosh N, Rainey H and Sandall J. Understanding how rapid response systems may improve safety for the acutely ill patient: learning from the frontline. BMJ Qual Saf. 2012 Feb;21(2):135-44. doi: 10.1136/bmjqs-2011-000147. Epub 2011 Oct 4. PMID: 21972419.

138. Martinez VA, Betts RK, Scruth EA, et al. The Kaiser Permanente Northern California Advance Alert Monitor Program: An Automated Early Warning System for Adults at Risk for In-Hospital Clinical Deterioration. Jt Comm J Qual Patient Saf. 2022 Aug;48(8):370-375. doi: 10.1016/j.jcjq.2022.05.005. PMID: 35902140.

139. McElroy T, Swartz EN, Hassani K, et al. Implementation study of a 5-component pediatric early warning system (PEWS) in an emergency department in British Columbia, Canada, to inform provincial scale up. BMC Emerg Med. 2019 Nov 27;19(1):74. doi: 10.1186/s12873-019-0287-5. PMID: 31771517; PMCID: PMC6880448.

140. McGaughey J, O'Halloran P, Porter S, et al. Early warning systems and rapid response to the deteriorating patient in hospital: A realist evaluation. J Adv Nurs. 2017 Dec;73(12):3119-3132. doi: 10.1111/jan.13367. Epub 2017 Aug 23. PMID: 28637090.

141. Menon VP, Prasanna P, Edathadathil F, et al. A Quality Improvement Initiative to Reduce "Out-of-ICU" Cardiopulmonary Arrests in a Tertiary Care Hospital in India: A 2-Year Learning Experience. Qual Manag Health Care. 2018 Jan/Mar;27(1):39-49. doi: 10.1097/QMH.0000000000000160. PMID: 29280907.

142. Miles I, Anderson M, Ren D, et al. Use of the Modified Early Warning Score by Medical-Surgical Nurses to Initiate the Rapid Response Team: Impact on Patient Outcomes. J Nurs Care Qual. 2023 Apr-Jun 01;38(2):171-176. doi: 10.1097/NCQ.0000000000000680. Epub 2022 Dec 3. PMID: 36729965.

143. Miller PJ. Case Study: Implementing Early Detection of Patient Deterioration in Medical and Surgical Units. Biomed Instrum Technol. 2016 Nov-Dec;50(6):439-446. doi: 10.2345/0899-8205-50.6.439. PMID: 27854491.

144. Mills D, Schmid A, Najajreh M, et al. Implementation of a pediatric early warning score tool in a pediatric oncology Ward in Palestine. BMC Health Serv Res. 2021 Oct 26;21(1):1159. doi: 10.1186/s12913-021-07157-x. PMID: 34702268; PMCID: PMC8549265.

145. Munroe B, Curtis K, Fry M, et al. Implementation evaluation of a rapid response system in a regional emergency department: a dual-methods study using the behaviour change wheel. Aust Crit Care. 2023 Sep;36(5):743-753. doi: 10.1016/j.aucc.2022.10.006. Epub 2022 Dec 7. PMID: 36496331.

146. Pain C, Green M, Duff C, et al. Between the flags: implementing a safety-net system at scale to recognise and manage deteriorating patients in the New South Wales Public Health System. Int J Qual Health Care. 2017 Feb 1;29(1):130-136. doi: 10.1093/intqhc/mzw132. PMID: 27920243.

147. Parshuram CS, Bayliss A, Reimer J, et al. Implementing the Bedside Paediatric Early Warning System in a community hospital: A prospective observational study. Paediatr Child Health. 2011 Mar;16(3):e18-22. doi: 10.1093/pch/16.3.e18. PMID: 22379384; PMCID: PMC3077313.

148. Paulson SS, Dummett BA, Green J, et al. What Do We Do After the Pilot Is Done? Implementation of a Hospital Early Warning System at Scale. Jt Comm J Qual Patient Saf. 2020 Apr;46(4):207-216. doi: 10.1016/j.jcjq.2020.01.003. Epub 2020 Jan 21. PMID: 32085952.

149. Raff L, Reilly K, Ratner S, et al. Building High-Performance Team Dynamics for Rapid Response Events in a US Tertiary Hospital: A Quality Improvement Model for Sustainable Process Change. Am J Med Qual. 2022 Sep-Oct 01;37(5):413-421. doi: 10.1097/JMQ.0000000000000057. Epub 2022 Apr 8. PMID: 35404304.

150. Rose MA, Hanna LA, Nur SA and Johnson CM. Utilization of electronic modified early warning score to engage rapid response team early in clinical deterioration. J Nurses Prof Dev. 2015 May-Jun;31(3):E1-7. doi: 10.1097/NND.0000000000000157. PMID: 25993463.

151. Rosen MJ, Hoberman AJ, Ruiz RE, et al. Reducing cardiopulmonary arrest rates in a three-year regional rapid response system collaborative. Jt Comm J Qual Patient Saf. 2013 Jul;39(7):328-36. doi: 10.1016/s1553-7250(13)39047-3. PMID: 23888644.

152. Samim SA, Singh A and Ravi P. Modified Early Warning System: Quality Improvement with the Help of Healthcare Failure Modes and Effect Analysis. Hosp Top. 2020 Jul-Sep;98(3):108-117. doi: 10.1080/00185868.2020.1788476. Epub 2020 Jul 7. PMID: 32633216.

153. Sebat F, Vandegrift MA, Childers S and Lighthall GK. A Novel Bedside-Focused Ward Surveillance and Response System. Jt Comm J Qual Patient Saf. 2018 Feb;44(2):94-100. doi: 10.1016/j.jcjq.2017.09.002. Epub 2018 Jan 3. PMID: 29389465.

154. Spiers L, Singh Mohal J, Pearson-Stuttard J, et al. Recognition of the deteriorating patient. BMJ Qual Improv Rep. 2015 Mar 25;4(1):u206777.w2734. doi: 10.1136/bmjquality.u206777.w2734. PMID: 26734344; PMCID: PMC4645861.

155. Sridhar S, Schmid A, Biziyaremye F, et al. Implementation of a Pediatric Early Warning Score to Improve Communication and Nursing Empowerment in a Rural District Hospital in Rwanda. Glob Health Sci Pract. 2020 Dec 23;8(4):838-845. doi: 10.9745/GHSP-D-20-00075. PMID: 33361246; PMCID: PMC7784060.

156. Stolldorf DP, Havens DS and Jones CB. Sustaining Innovations in Complex Health Care Environments: A Multiple-Case Study of Rapid Response Teams. J Patient Saf. 2020 Mar;16(1):58-64. doi: 10.1097/PTS.0000000000000239. PMID: 26756725; PMCID: PMC4940275.

157. Suhr K, Steen C, Albrecht-Thompson R and Williams J. NEWS Scoring System: Use in Hematologic Malignancies and Cellular Therapeutics Patient Populations. Clin J Oncol Nurs. 2020 Apr 1;24(2):E21-E27. doi: 10.1188/20.CJON.E21-E27. PMID: 32196014.

158. Tanguay T and Bartel R. Implementation of a unique RRT model in a tertiary care centre in Western Canada. Can J Crit Care Nurs. 2017 May;28(1):34-37. PMID: 29465178.

159. van der Fluit KS, Boom MC, Brandão MB, et al. How to implement a PEWS in a resource-limited setting: A quantitative analysis of the bedside-PEWS implementation in a hospital in northeast Brazil. Trop Med Int Health. 2021 Oct;26(10):1240-1247. doi: 10.1111/tmi.13646. Epub 2021 Jul 21. PMID: 34192384; PMCID: PMC8596539.

160. Vandegrift MA, Granata R, Totten VY, et al. Review of 20 Years of Continuous Quality Improvement of a Rapid Response System, at Four Institutions, to Identify Key Process Responsible for Its Success. Crit Care Explor. 2021 Aug 10;3(8):e0448. doi: 10.1097/CCE.0000000000000448. PMID: 34396140; PMCID: PMC8357252.

161. Watson D and Carberry M. Nurses' experiences of recording vital signs electronically: a pilot study. Nursing Times, 2021. 117(2): p. 55-58.

162. Williams KM. Above PAR Care: Implementation of a Failure- to-Rescue Strategy. PACEsetterS 8(1):p 31-35, January 2011. | DOI: 10.1097/01.JBI.0000395925.85130.7e

163. Winterbottom FA and Webre H. Rapid Response System Restructure: Focus on Prevention and Early Intervention. Crit Care Nurs Q. 2021 Oct-Dec 01;44(4):424-430. doi: 10.1097/CNQ.0000000000000379. PMID: 34437321.

164. Wood SD, Candeland JL, Dinning A, et al. Our approach to changing the culture of caring for the acutely unwell patient at a large UK teaching hospital: A service improvement focus on Early Warning Scoring tools. Intensive Crit Care Nurs. 2015 Apr;31(2):106-15. doi: 10.1016/j.iccn.2014.11.006. Epub 2015 Jan 17. PMID: 25604030.
